# Regulation and Mechanism of Deleted in Breast Cancer‐1 on Dendritic Cell Function in Systemic Lupus Erythematosus

**DOI:** 10.1002/mco2.70581

**Published:** 2026-01-14

**Authors:** Ze Xiu Xiao, Rongzhen Liang, Yan Liu, Changyuan Huang, Qiannan Fang, Xiaojiang Hu, Julie Wang, Nancy Olsen, Dehua Wu, Song Guo Zheng

**Affiliations:** ^1^ Department of Anesthesiology Songjiang Hospital affiliated to the Shanghai Jiao Tong University School of Medicine Shanghai China; ^2^ Department of Immunology the School of Cell and Gene Therapy Songjiang Research Institute Songjiang Hospital affiliated to the Shanghai Jiao Tong University School of Medicine Shanghai China; ^3^ Department of Clinical Immunology The Third Affiliated Hospital of Sun Yat‐Sen University Guangzhou China; ^4^ Department of Rheumatology The Third Affiliated Hospital of Sun Yat‐Sen University Guangzhou China; ^5^ The First Dongguan Affiliated Hospital Guangdong Medical University Dongguan China; ^6^ Medical Research institute Guangdong Provincial People's Hospital (Guangdong Academy of Medical Science) Southern Medical University Guangzhou China; ^7^ Department of Medicine Penn State College of Medicine Hershey Pennsylvania USA

**Keywords:** autoimmune disease, deleted in breast cancer‐1, dendritic cells, STAT5, systemic lupus erythematosus

## Abstract

Systemic lupus erythematosus (SLE) is a chronic autoimmune disease affecting multiple organs and involving both innate and adaptive immunity. Dendritic cells (DCs) play a crucial role in linking innate and adaptive immune responses, and therefore they deeply participate in the initiation and development of SLE. Deleted in breast cancer‐1 (DBC1) is a negative regulator of deacetylase SIRT1 (the mammalian homolog of silent information regulator 1) and involves in tissue inflammation. Roles of DBC1 in immune cells remain largely unknown, especially in DCs. We here identified that DBC1 is upregulated in activated DCs, and DBC1 deficiency weakened DC maturation while promoting B7‐H1 expression. DC conditional knockout of DBC1 ameliorated murine lupus pathology by decreasing autoantibodies, complement C3, plasma cells, and follicular T helper (Tfh) cells, whereas promoting regulatory T‐cell development. We further demonstrated that *Dbc1^−/−^* DC lowered proinflammatory cytokine secretion such as IL‐4, IL‐6, and IL‐12, and reduced signal transducer and activator of transcription 5 (STAT5) signal. With STAT5 overexpression, the protective effect by *Dbc1^−/−^* DC was abolished in the lupus model. Therefore, targeting the DBC1‐STAT5 axis in DCs diversifies the therapeutic strategies for SLE.

## Graphical abstract

LPS‐stimulated DC activation upregulated DBC1. DBC1‐deficient DC ameliorated murine SLE symptoms. DBC1 deficiency suppressed immune responses in DC. DBC1 deficiency lowered STAT5 expression and subsequent phosphorylation. STAT5 overexpression abolished murine SLE remission primed by DBC1 deficiency.

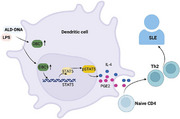

## 1 Introduction

Systemic lupus erythematosus (SLE) is a complex autoimmune disease emerging with systemic inflammation and multi‐organ damage, predominantly affecting childbearing women [1, 2]. The pathogenesis of SLE involves a breakdown of immune tolerance, leading to the production of autoantibodies, immune complex deposition, and subsequent tissue injury [3, 4, 5]. A significant pathophysiological feature of SLE is abundant autoantibody production, among which the anti‐double stranded DNA (dsDNA) antibody serves as a key SLE diagnostic marker and correlates with disease severity, particularly in lupus nephritis (LN), a major cause of morbidity and mortality in SLE patients [6, 7]. Despite advances in treatment, including glucocorticoids, immunosuppressive drugs and biological agents, SLE remains incurable nowadays. Underscoring the need for a deeper understanding of its pathogenesis mechanisms will guide more effective and targeted therapeutic strategies.

Dendritic cells (DCs) play a pivotal role in SLE pathogenesis by bridging innate and adaptive immunity, promoting autoantibody production and sustaining chronic inflammation [8, 9, 10]. Elevated DC levels in the peripheral blood of SLE patients, along with their heightened activation status, contribute to immune tolerance loss and the perpetuation of autoimmune responses. Studies have revealed that type I interferons (IFN‐α) in SLE sera induced DCs differentiation and activation, further exacerbating disease progression [11, 12, 13]. Our previous research using a murine SLE model induced by bone marrow–derived DC activated by lymphocytes‐derived DNA (BMDC‐ALD‐DNA) demonstrated that DCs are critical in SLE development, with co‐stimulatory factors, transcription factors, and dietetic factors modulating their functions [14, 15, 16]. Moreover, B7‐H4 expression on DCs also enhances regulatory T cell (Treg) differentiation and ameliorates SLE symptoms, highlighting the therapeutic potential of modulating DC–Treg interactions [14, 17]. Currently, therapies targeting antigen presentation of DCs has achieved clinical effects in SLE. Clinical trials targeting antibody binding of blood dendritic cell antigen 2 (BDCA2), which expressed exclusively on plasmacytoid dendritic cells, alleviated skin lesions and displayed reduction in the number of swollen and tender joints than placebo [18, 19].

Deleted in Breast Cancer‐1 (DBC1), also known as cell cycle and apoptosis regulator 2 (CCAR2), is a multifunctional transcription factor involved in apoptosis, metabolism, DNA repair, and tumorigenesis [20]. The roles of DBC1 in cancer have been studied, for example, DBC1 is able to inhibit breast cancer progression by suppressing the deacetylase activity of sirtuin‐type deacetylase (SIRT1), a NAD^+^‐dependent deacetylase involved in aging and stress responses, and promoting cellular apoptosis [21]. DBC1 has also been reported to interact with poly ADP‐ribose polymerase (PARP1) and participates in DNA repair process [22]. Conversely, another study reported that DBC1 acts as a survival factor for breast cancer cells by interacting with estrogen receptor (ERα) through its hormone‐binding domain, but with no effect on the mRNA level of ERα [23]. As promoting tumor formation in some cases while suppressing tumor growth or survival in others, the physiological functions of DBC1 in vivo remain to be determined [24]. Recently, emerging evidence suggests that DBC1 also regulates immune cell function by inhibiting B‐cell activation via suppressing nuclear factor kappa‐B (NF‐kB) signaling, and promoting FOXP3 degradation in Tregs, reducing their suppressive capacity [25, 26]. Given the critical roles of both B cells and Tregs in SLE, we hypothesize that DBC1 may also influence DC function and contribute to SLE pathogenesis.

To investigate this possibility, we generated a conditional knockout mouse strain (*Cd11c^cre^Dbc1^fl/fl^*) with DC‐specific deletion of Dbc1. These mice model exhibited no significant differences in immune cell development compared to wide‐type (WT) mice but displayed attenuated SLE symptoms, including reduced autoantibody production and LN severity in an SLE mouse model. Mechanistically, we ruled out that DBC1 deficiency in DCs impairs STAT5 signaling, a pathway critical for DC activation and pro‐inflammatory responses. Our findings suggest that DBC1 is upregulated in activated DCs and facilitates the expression and phosphorylation of STAT5, which in turn promotes DC‐mediated inflammation and aggravates SLE initiation and progression. This study not only identifies DBC1 as a novel regulator of DC function in SLE but also provides a potential therapeutic target for modulating DC‐driven autoimmunity.

## 2 Results

### 2.1 DBC1 Participates in the Activation but Not Development of Dendritic Cells

**FIGURE 1:**
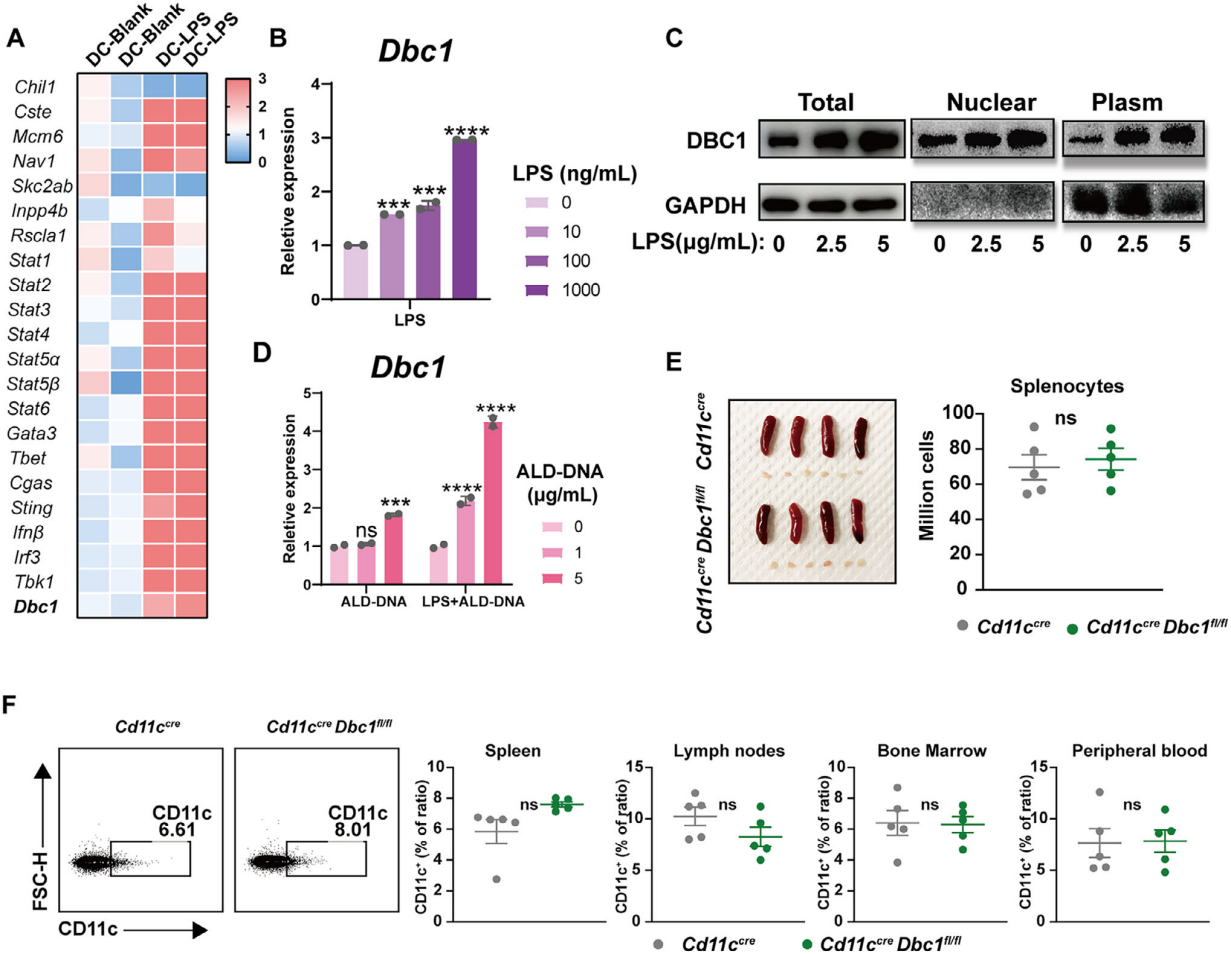
DBC1 participates the activation but not development of dendritic cells. (A) Transcriptional alteration of DC after the stimulation of LPS. (B) Relative expression of DBC1 after the different LPS concentration stimulation. (C) Relative expression of DBC1 after the different concentration of ALD‐DNA with or without LPS (10 ng/mL) stimulation. (D) Translational level of DBC1 altered after LPS stimulated DCs. (E) Gross look and total splenocytes of *Cd11c^cre^* and *Cd11c^cre^ Dbc1^fl/fl^* mice. (F) The ratio of DC of different tissues from the *Cd11c^cre^* and *Cd11c^cre^ Dbc1^fl/fl^* mice. The values are presented as the mean ± SEM. The results are the representative from three independent experiments. ns means no significance, **p < 0.005, ***p < 0.0005, and ****p < 0.0001 using nonparametric *t* tests or one‐way ANOVA.

Lipopolysaccharide (LPS) is a commonly used DC activator. Our study revealed that LPS not only upregulated a variety of transcription factors, including the well‐known JAK‐STATs, but also significantly increased the transcriptional level of DBC1 in DCs (Figure 1A). The dose‐dependent stimulation experiments demonstrated a positive correlation between DC activation and DBC1 expression (Figure 1B), and western blots analysis further confirmed the overexpression of DBC1 in both the nucleus and cytoplasm of DCs upon activation (Figure 1C). Additionally, DCs co‐cultured with ALD‐DNA, previously used for establishing a murine SLE model, also exhibited increased DBC1 expression. Notably, the combination of low‐dosage LPS and ALD‐DNA stimulated the level of DBC1 in DCs furtherly (Figure 1D), suggesting that DBC1 is involved in the activation process of DCs.

To elucidate the specific role of DBC1 in DCs, we generated a Dbc1 floxed mouse strain Dbc1^fl/fl^ and crossed it with *Cd11c^cre/cre^* mouse to create DC‐specific DBC1 knockout mice (Figure S1A). The resulting *Cd11c^cre/cre^ Dbc1^fl/fl^* mice were genotyped to confirm the presence of the floxed *Dbc1* allele and the Cre recombinase transgene. These mice appeared healthy and exhibited no significant differences compared to *Cd11c^cre/cre^* controls in terms of the gross morphology of immune tissues, including spleen and lymph nodes, or in the total splenocyte numbers (Figure 1E). We further examined the ratio of various immune cells in the spleen, lymph nodes, bone marrow, and peripheral blood. Our results showed that the development of DCs (Figure 1F) and the other immune cells, including T cells, B cells, macrophages, neutrophils, and NK cells (Figure S1B), was not affected by the conditional knockout of DBC1. Furthermore, the subsets of DCs, such as DC1 or DC2, as well as the expression levels of maturation and activation markers on DCs, were all within the normal range (Figure S1C). Taken together, the above data demonstrate that DBC1 is associated with the activation of DCs but does not influence their development.

### 2.2 DBC1‐Deficient DCs Ameliorate the Symptoms of SLE

**FIGURE 2:**
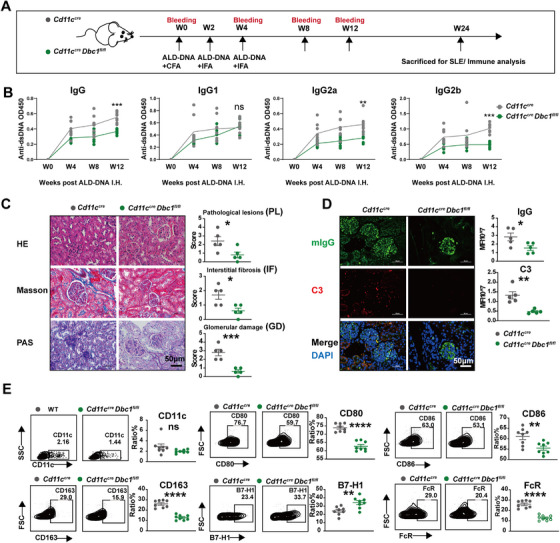
DBC1‐deficient in DCs ameliorate the symptoms of murine lupus. (A) Experiment schedule of ALD‐DNA immunization of *Cd11c^cre^ and Cd11c^cre^ Dbc1^fl/fl^* mice. (B) Anti‐dsDNA antibody of constructed SLE murine model. (C) HE, Masson, and periodic acid‐Schiff (PAS) staining of renal sections and the pathological scores of constructed SLE murine model. (D) Immunofluorescence showing the deposition of IgG and C3 in renal sections from constructed SLE murine model. (E) The functional flow cytometry analysis of dendritic cell from peripheral blood of SLE murine model mice. The values are presented as the mean ± SEM. The results are the representative from three independent experiments. ns means no significance, **p < 0.005, ***p < 0.0005, and ****p < 0.0001 using nonparametric *t* tests or two‐way ANOVA.

Xiong and colleagues previously reported that immunization of recipient mice with activated syngeneic lymphocyte‐derived DNA (ALD‐DNA) could successfully induce murine SLE syndromes [27] and we have also verified this model. In the current study, we constructed an SLE model using DC‐specific DBC1 knockout mice (*Cd11c^cre/cre^ Dbc1^fl/fl^*) (Figure 2A). As shown in Figure 2B, the levels of anti‐dsDNA antibody total IgG, as well as IgG1, IgG2a, and IgG2b subtypes, were significantly lower in *Cd11c^cre/cre^ Dbc1^fl/fl^* mice, compared to wild‐type (WT) controls. Additionally, LN manifestations, including the kidney pathological lesions, interstitial fibrosis, and glomerular damage (GD), showed obvious amelioration in DC‐specific DBC1 knockout mice (Figure 2C). The immuno‐complex deposition of IgG and complement C3 in the kidneys was also reduced compared to WT controls (Figure 2D). While the counts of DCs in peripheral blood did not show significant differences between the two groups, the maturation makers (CD80, CD86) and inflammatory functional factors (CD163, FcR) on DC were distinctly decreased in *Cd11c* ^cre/cre^ *Dbc1* ^fl/fl^ mice, and the co‐inhibitory factor B7‐H1 was increased (Figure 2E). Notably, DBC1 conditional knockout in DCs did not alter the expression of CD40, MHCII, and CCR7, which represents DC maturation and activation (Figure S2A). In addition, DBC1 deficiency in DCs did not alter B cell or plasmacyte populations but reduced total CD3^+^ T cells. Within this T‐cell compartment, the CD4^+^ subset remained unchanged while the CD8^+^ population increased (Figure S2B). Since B cells have the critical role in SLE progression, especially antibody production, we detected costimulatory, regulatory, activated, and antibody response–related markers. Interestingly, DC conditional knockout of DBC1 markedly promoted B7‐H1 (namely, PD‐L1) expression in B cells, accompanied by unaltered expression of CD80, CD69, CD40, CD5, IgG, and IgM (Figure S2C). These results highlight the role of DBC1 in DC maturation and proinflammatory function.

**FIGURE 3:**
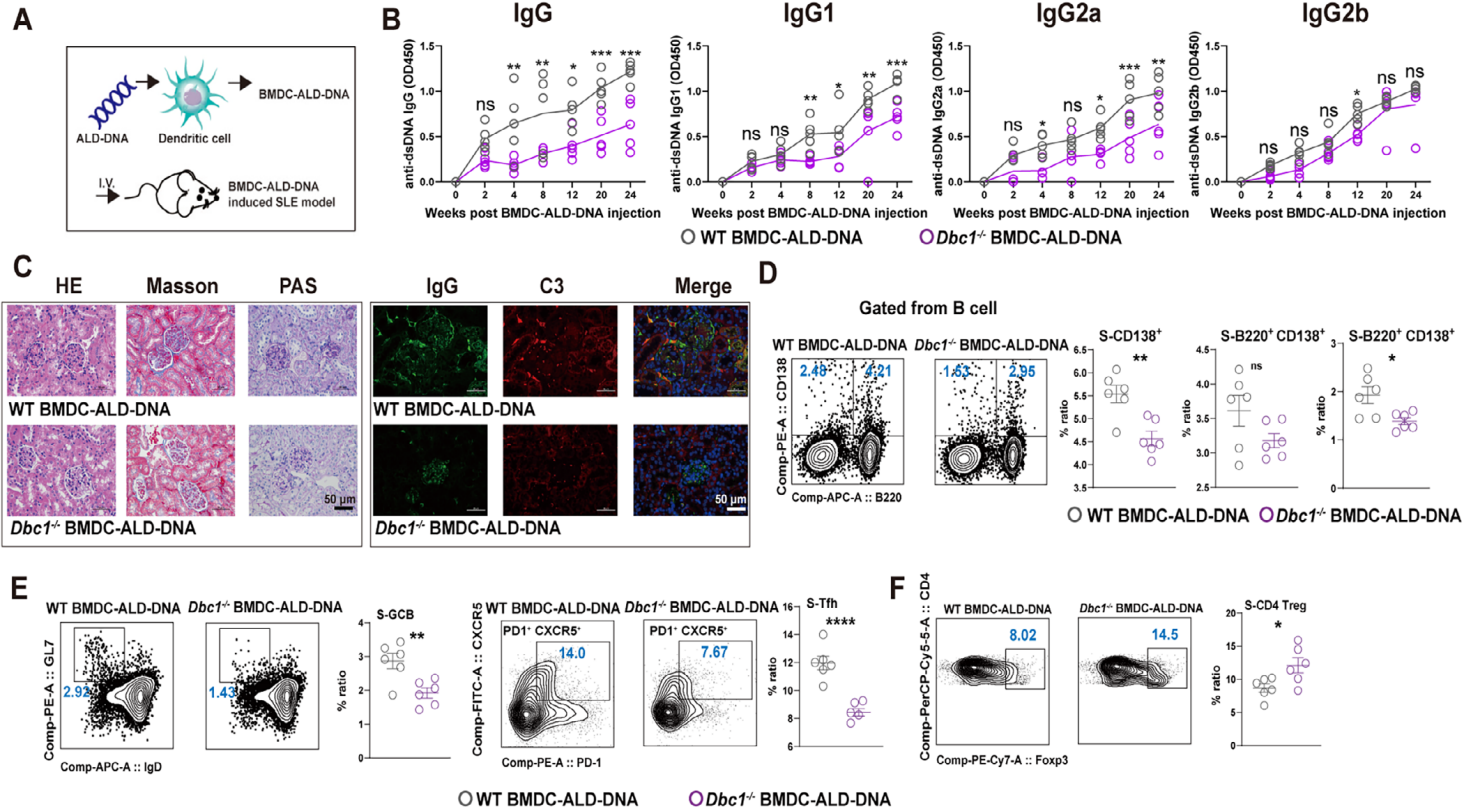
DBC1 knockout BMDC‐ALD‐DNA initiates milder murine lupus manifestations. (A) Experiment schedule of BMDC‐ALD‐DNA constructed SLE murine model. (B) Anti‐dsDNA antibody of constructed SLE murine model. (C) HE, Masson, and periodic acid‐Schiff (PAS) and Ig deposition; C3 deposition staining of renal sections; and the pathological scores of constructed SLE murine model. (D) The plasmacytes from spleen of constructed SLE murine model. (E) GCB (B220^+^ GL7^+^ FAS^+^) and Tfh (CD4^+^ CXCR5^+^ PD1^+^) from spleen of constructed SLE murine model. (F) Treg cells from the spleen and lymph nodes of constructed SLE murine model. The values are presented as the mean ± SEM. The results are the representative from three independent experiments. ns means no significance, **p < 0.005, ***p < 0.0005, and ****p < 0.0001 using nonparametric *t* tests or two‐way ANOVA.

We previously established a murine SLE model using BMDCs loaded with ALD‐DNA (Figure 3A) [14]. In this study, the *Dbc1^−/−^* BMDC exhibited weaker processing of ALD‐DNA and induced milder SLE manifestations compared to WT BMDCs (Figure 3B,C). The ratio of antibody‐producing plasmacytes was decreased in SLE mice constructed with the *Dbc1^−/−^* BMDCs (Figure 3D). Moreover, cell percentages of the germinal center B cells (GCB) and follicular T helper cells (Tfh), which are critical for plasma cell differentiation and Ig production [28, 29, 30], were also reduced in the *Dbc1^−/−^* mice (Figure 3E). Tregs, which are responsible for modulating immune responses and autoimmunity [31, 32], were significantly increased in the spleen and lymph nodes of the *Dbc1^−/−^* BMDCs‐ALD‐DNA constructed SLE mice (Figure 3F). Collectively, these findings demonstrate that DBC1 deficiency impairs DCs maturation and function, thereby attenuating the severity of murine SLE.

### 2.3 *Dbc1^−/−^* DC Exerts Lower Immune Responses Than Wide‐Type DC In Vitro

**FIGURE 4:**
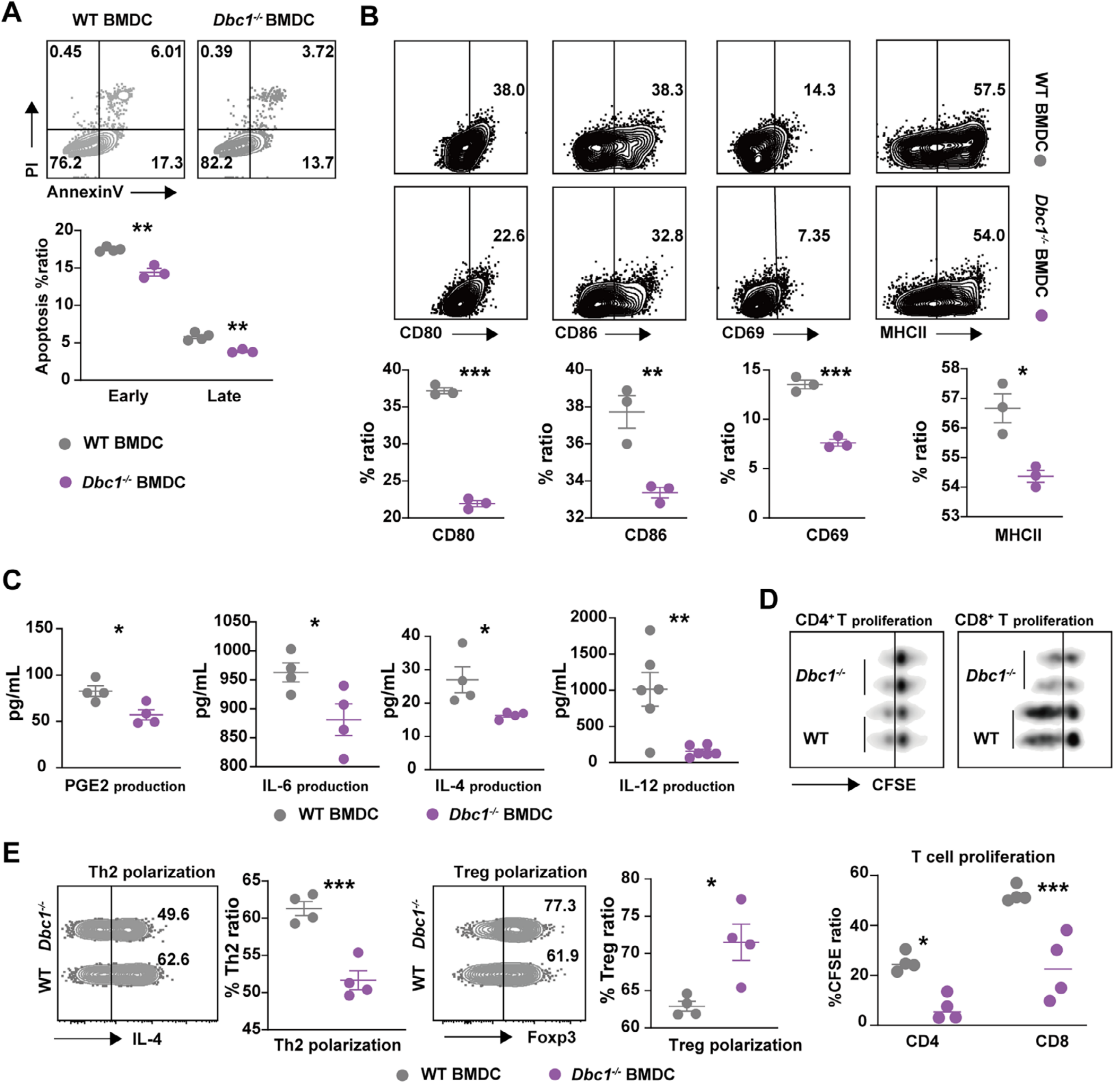
DBC1 knockout DCs exert lower immune responses than wide type. (A) The apoptosis level of WT or *Dbc1^−/−^* DCs. (B) The maturation and activation markers of WT or *Dbc1^−/−^* DCs after the stimulation of ALD‐DNA. (C) The PGE2, IL‐6, IL‐4, and IL‐12 production of WT or *Dbc1^−/−^* DCs. (D) T‐cell proliferation initiated by WT or *Dbc1^−/−^* DCs. (E) The Th2 and Treg cells polarization from the WT or *Dbc1^−/−^* DCs priming. The values are presented as the mean ± SEM. The results are the representative from three independent experiments. ns means no significance, **p < 0.005, ***p < 0.0005, and ****p < 0.0001 using nonparametric *t* tests.

As shown in Figure 1F, DBC1 deficiency did not influence the development of DCs either in vivo and in vitro (Figure S3A). Given that DBC1 has been reported as an apoptosis regulatory factor in tumor cells [21], we investigated its role in DC apoptosis. We noted that DBC1 knockout in DCs decreased the apoptosis level compared to WT controls (Figure 4A). The expression of CD80, CD86, CD69, and MHCII on BMDCs following stimulation with LPS or ALD‐DNA was significantly lower in *Dbc1^−/−^* DCs than that in WT DCs, demonstrating that the maturation and activation of DCs were destructed as the DBC1 deficiency (Figure 4B and Figure S3C).

In addition to maturation and activation, DCs play a crucial role in cytokines or chemokines secretion. As shown in Figure 4C, the production of prostaglandin E2 (PGE2), IL‐6, IL‐4, and IL‐12 was significantly decreased in *Dbc1^−/−^* DCs compared to that in WT DCs (Figure 4C). In contrast, the levels of TNF‐α, IFN‐γ, IFN‐β, IL‐10, and CCL17 displayed no obvious differences between the two groups (Figure S3B). The polarization of different T‐cell phenotypes from naïve T cells is dependent on signals provided by DCs or other antigen‐presenting cells [33]. IL‐4 and PGE2 are essential for Th2 cells polarization and development [34], while IL‐6 is critical for Th17 differentiation [35]. Consistent with the reduced production of PGE2, IL‐4, and IL‐6 in DBC1‐deficient DCs, we observed that Th2 and Th17 polarization was significantly reduced when polarizing with mitomycin‐C treated DBC1‐deficient DCs, whereas Treg priming was enhanced. Th1 differentiation was not affected, as the IFN‐γ production remained unchanged between WT and *Dbc1^−/−^* DCs (Figure 4D and Figure S3D).

Another important function of DC is the priming of T‐cell proliferation and the potential of T helper cells polarization [16], We noted that DBC1 deficiency in DCs induced weaken proliferation of both CD4^+^ and CD8^+^ T cells (Figure 4D), and *Dbc1^−/−^* DC exhibited reduced Th2 cell polarization, but enhanced Treg cell polarization (Figure 4E), which is consistent with the increased Treg cell population in the DC‐specific DBC1 knockout SLE model mice (Figure 3F). In contrast, Th1 and Th17 polarization showed no obvious differences from WT and *Dbc1^−/−^* DC (Figure S3D). Th2 cells are known to play vital roles in the pathogenesis of SLE [36], and the qualitative deficiencies in Treg cells contribute to immune dysregulation, leading to the development and deterioration of SLE [37]. Taken together, our findings demonstrate that DBC1 deficiency in DCs results in reduced cytokines production, decreased T cells proliferation, impaired Th2 cells polarization and enhanced Treg cells polarization. These characteristics are likely responsible for the amelioration of SLE development observed in DBC1‐deficient mice.

### 2.4 DBC1 Regulates the Inflammatory Factor of DCs Through STAT5 Pathway

**FIGURE 5:**
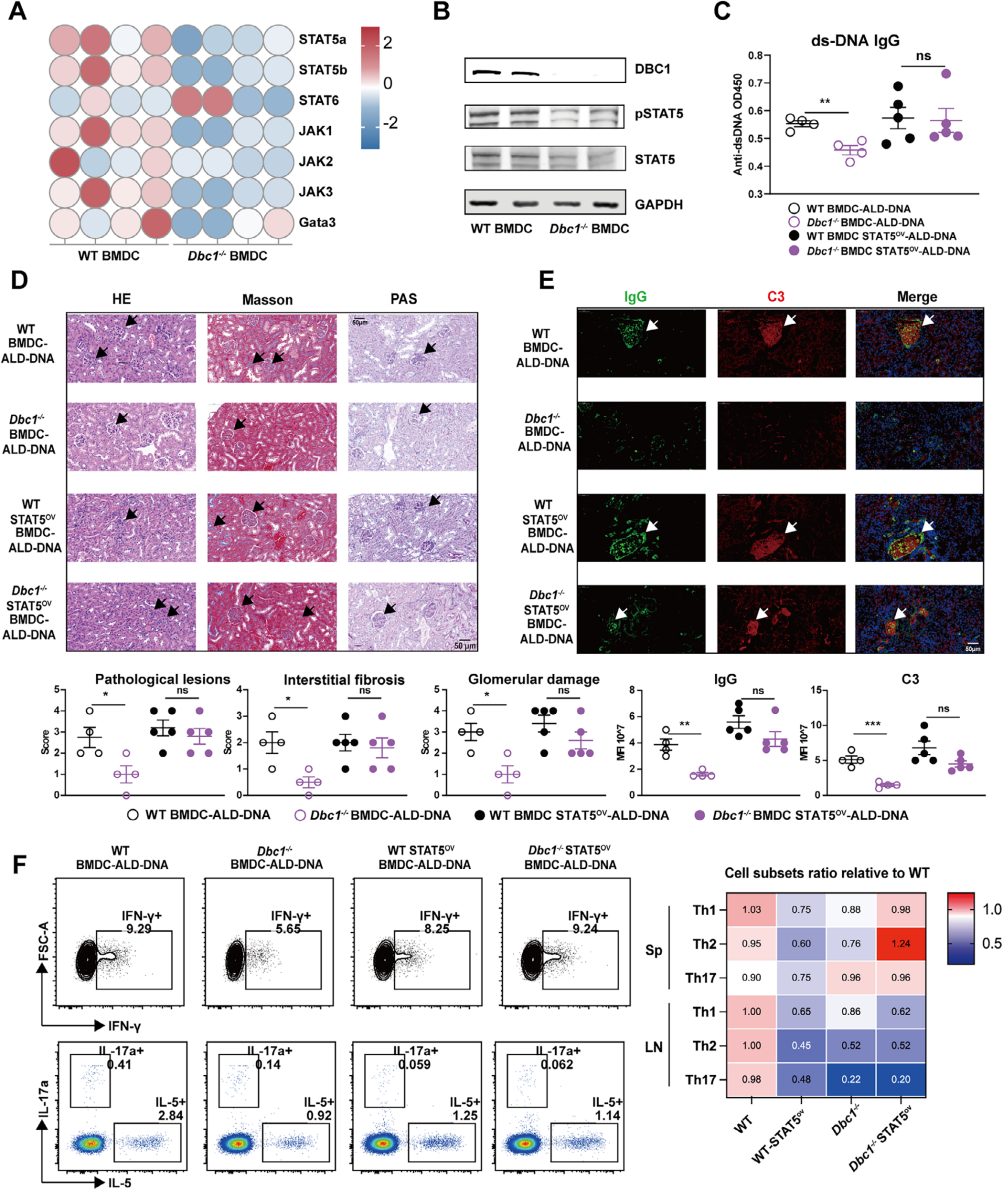
DBC1 regulates the inflammatory factor of DCs through STAT5 pathway. (A) The Th2‐related transcript factors QPCR array of WT and *Dbc1^−/−^* BMDCs. (B) The total STAT5 and phosphate STAT5 protein levels of WT and *Dbc1^−/−^* BMDCs. (C) The anti‐ds‐DNA IgG of different four group BMDC‐ALD‐DNA induced SLE mice. (D) The HE, Masson, and PAS‐stained kidney sections of different four groups of BMDC‐ALD‐DNA induced SLE mice. (E) The IgG and C3 deposition IF staining of different four groups of BMDC‐ALD‐DNA induced SLE mice. (F) The Th cell subsets of spleen and LN from different four groups of BMDC‐ALD‐DNA induced lupus mice. The values are presented as the mean ± SEM. The results are the representative from three independent experiments. ns means no significance, **p < 0.005, ***p < 0.0005, and ****p < 0.0001 using one‐way ANOVA.

As above described, DBC1 deficiency in DCs significantly reduced IL‐4 secretion (Figure 4C) and impaired DC‐primed Th2 polarization (Figure 4E). These indicate that DBC1 may be involved in the Th2‐specific transcription factor signaling pathway, such as the STAT5 pathway [38]. To investigate this possibility, we performed RNA qPCR array analysis on WT and *Dbc1^−/−^* BMDCs and observed a downregulation of STAT5 mRNA in *Dbc1^−/−^* BMDCs (Figure 5A). Additionally, both total and phosphorated protein levels of STAT5 were reduced in DBC1‐deficient DCs (Figure 5B).

To further explore the role of STAT5 in the amelioration of murine SLE by DBC1 deficiency, we overexpressed STAT5 in BMDCs using a STAT5β overexpression plasmid (Figure S4). WT and *Dbc1^−/−^* BMDCs were pretreated with STATβ overexpression plasmid and then stimulated with ALD‐DNA. These BMDC‐ALD‐DNA were subsequently used to induce lupus in recipient C57BL/6 mice. The results showed that STAT5β‐overexpressing *Dbc1^−/−^* BMDCs induced similar levels of anti‐dsDNA antibodies compared to WT BMDC‐ALD‐DNA group mice (Figure 5C and Figure S5A). Moreover, the LN manifestations including severe pathological lesions, interstitial fibrosis and GD, were exacerbated in mice treated with STAT5β‐overexpressing *Dbc1^−/−^* BMDCs (Figure 5D). Consistent with these findings, the deposition of IgG and complement C3 also demonstrated similar changes after the STAT5 signal compensating (Figure 5E), suggesting that DBC1 regulates the responses of DC depending on STAT5 pathway.

Mechanistically, the restoration of STAT5 signaling rescued Th2 differentiation in SLE model mice (Figure 5F). Given that DBC1 deficiency reduced Th2 polarization in vitro, the enhanced Th2 response after STAT5 compensation in the *Dbc1^−/−^* BMDC‐ALD‐DNA likely contributed to the exacerbated SLE syndromes. In addition, the numbers of B cells and GCB cells were also increased in the spleen of mice with STAT5 signaling restoration (Figure S5B–D). Conclusively, these results imply that DBC1 regulates DC function through the STAT5 pathway, highlighting an important role of DBC1‐STAT5‐Th2 axis in the development of murine lupus. Targeting this axis may provide a potential therapeutic strategy for SLE and other autoimmune diseases.

### 2.5 DBC1 Regulates Inhibitory and Proinflammatory Signals via Targeting Multiple Molecules

To further identify how DBC1 modulates DC function, we also used RNA‐seq to analyze underlying molecules alteration. Principal component analysis (PCA) showed a near location of unstimulated WT with *Dbc1^−/−^* BMDCs but a distinct position between LPS‐stimulated WT and *Dbc1^−/−^* BMDCs, suggesting that DBC1 deficiency in DCs had narrow influence under steady stage but remarkably change DC maturation and activation upon stimulation (Figure S6A). Differentially expressed genes showed that DBC1 deficiency upregulates inhibitory receptors including *Tgfbr2* and *Il10ra*, whereas it downregulates proinflammatory‐related genes such as *Cd69*, *Cxcl10*, and *Il12a* (Figure S6B). KEGG analysis revealed significant pathway changes in cytokine signaling (Figure S6C) and Reactome analysis showed PD1/PD‐L1 signal changes (Figure S6D), indicating DBC1 knockdown regulates DC interaction with other cells. GSEA analysis revealed DBC1 knockdown positively correlates with cytokine‐cytokine receptor interaction, primary immunodeficiency, and cell‐adhesion molecules. We noted the trend that DBC1 deficiency leads to decreased SIRT1‐negatively regulated expression, which accords with the previous study that DBC1 inhibits SIRT1. Thus, we consider that DBC1 regulates DC function at least partially depending on SIRT1 relative pathways (Figure S6E). It is reported that thymic stromal lymphopoietin (TSLP) activated STAT5 activation in DCs [39], and whether DCs secretes TSLP to modulate STAT5 activation remains unknown. Though sources of TSLP are diverse, epithelial cells and stromal cells in the lungs, skin, and gastrointestinal tract are the primary sources; DCs can also produce TSLP upon stimulation. Normally, extracellular TSLP binds to heterodimeric receptor of TSLPr and IL‐7r and cause STAT5 activation in DCs. RNA‐seq results of WT and *Dbc1^−/−^* BMDCs showed no difference in mRNA level of *Tslp*, *Tslpr*, and *Il7r* between WT and *Dbc1^−/−^* BMDC (Figure S7A), suggesting DBC1 regulates STAT5 mostly independent of TSLP and TSLP receptor.

To explore whether DBC1 expression in DCs correlates with SLE, we compared the DBC1 mRNA level of human DCs from healthy donors and SLE patients. However, a bulk RNA‐seq dataset (E‐GEAD‐397) and two single‐cell RNA‐seq datasets (GSE135779 and GSE174188) showed no remarkable difference in *DBC1* expression in total DCs within SLE (Figure S7B–D). Nevertheless, these datasets based on mRNA level assessment may not rule out post‐translational modifications and protein degradations; it deserves a future study focusing on whether DBC1 function alteration in DCs is related to the patients with SLE.

**FIGURE 6:**
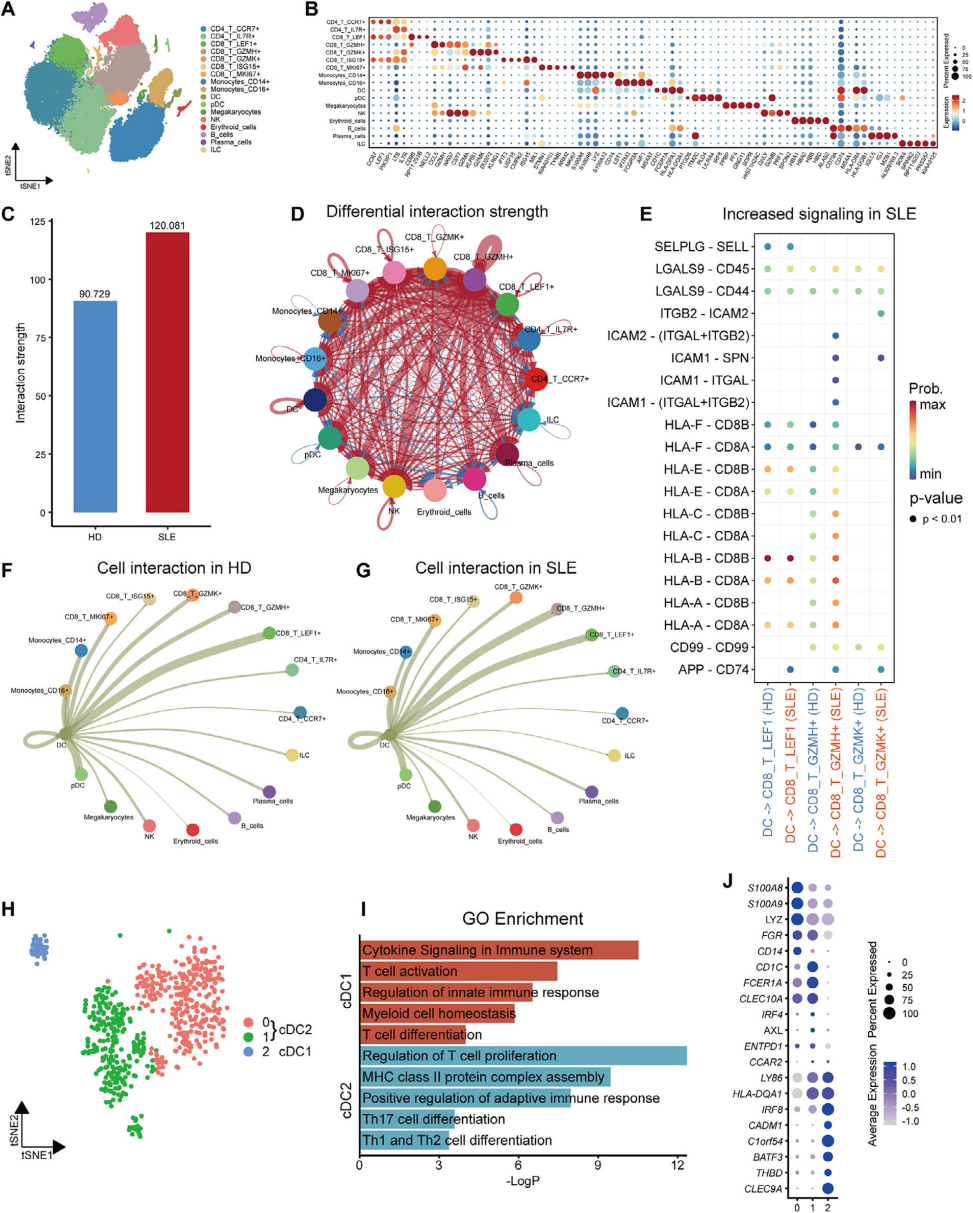
SLE emerges with enhanced DC‐T cell interaction. (A) A UMAP plot representing 17 clusters across PBMCs from five healthy adults (HD) and seven SLE patients (SLE) (data from GSE135779). The putative identity of each cluster was specified on the basis of (B). DC, dendritic cell; pDC, plasmacytoid DC; NK, natural killer. (B) Cluster annotation. The dot plot represents expression values of selected genes (*x* axis) across each cluster (*y* axis). The dot size indicates the percentage of cells expressing the marker of interest. The color intensity represents the mean expression within expressing cells. (C) Interaction strength of total clusters. (D) Differential interaction strength among clusters. Blue line means interaction decrease; red line represents interaction increase. Line thickness indicates interaction strength. (E) Increased signaling in SLE. The dot means statistical significance. The color intensity represents the mean interaction strength. (F) Cell interaction between DC and other clusters in HD. (G) Cell interaction between DC and other clusters in SLE. The line thickness represents interaction strength (F and G). (H) Subclusters of DC cluster. (I) GO enrichment of DC subclusters. (J) Annotation of DC subcluster. The dot plot represents expression values of selected genes (*y* axis) across each cluster (*x* axis). The dot size indicates the percentage of cells expressing the marker of interest. The color intensity represents the mean expression within expressing cells.

To investigate DC transcriptional features and interaction patterns with others immune cells, we further analyzed peripheral cell populations of 12 individuals containing five healthy adults (HD) and seven SLE patients from the single‐cell RNA seq datasets GSE135779. After relabeling the uniform manifold approximation and projection (UMAP), a conventional DC cluster and a plasmacytoid DC (pDC) cluster were separated and conventional DC highly characterized by highly expressed HLA‐related genes (Figure 6A,B). Immune cells of SLE showed higher interaction compared to HD (Figure 6C). We observed that DC interacted with all kinds of immune cells but evidently with CD8^+^ T cells (Figure 6D). We next sought to determine which interaction signal from DC was increased in SLE, and found increased DC‐CD8^+^ T cell interaction was mostly enriched in antigen presentation between DC‐CD8^+^ GZMH^+^ T cell (Figure 6E–G). The repertoire‐restricted cytotoxic GZMH^+^ CD8^+^ T cells were expanded in SLE with highly expressed cytotoxic, exhaustion, and interferon‐stimulated genes signatures [40]. To investigate DBC1 level in different DC subsets, DC subsets were distinctly separated into three clusters, in which cluster 0 and 1 belonged to cDC2, and cluster 2 was enriched in cDC1 (Figure 6H). Conventional type 1 dendritic cells (cDC1) function on antigen cross‐presentation to prime CD8^+^ T cells, while cDC2 cells perform an action on priming CD4^+^ T cells [41]. According to that, cDC2 cells mainly function on priming CD4^+^ T‐cell responses, and DBC1 expression on cDC2 may differently regulate CD4^+^ T cell. A recent study reported two distinct cDC2 lineages that had distinct function during immune responses [42], and our results displayed similar clusters (Figure 6I). cDC2 with highly expressed T‐bet, Runx3, and SREBF2 had lower CD86 and exerted anti‐inflammatory effects. Conversely, cDC2 with high level of C‐type lectin receptor CLEC10A promoted pro‐inflammatory responses [42]. We noticed the two cDC2 clusters had different DBC1 (namely CCAR2) expression levels, though their expression level was low. cDC2 with lower DBC1(cluster 0) emerged a phenotype of CD14^hi^ CD1c^low^ that was reported to share features with monocytes with high expression of Ca^2+^ binding S100 protein S100A8/9 and suppress antigen‐specific T‐cell response [43]. DBC^1ow^ cDC2 had a low level of HLADQA1, CD1C, and FCR1A compared with DBC1^hi^ cDC2, suggesting a mild antigen‐presenting ability (Figure 6J). Collectively, DBC1 expression level in human DCs represents pro‐inflammatory and anti‐inflammatory status in priming immune responses; therefore, targeting DBC1 shows potential in clinical translation.

## 3 Discussion

DBC1 is a pleiotropic regulatory protein in modulating apoptosis, metabolism, and transcription factors [44, 45]. The deacetylase SIRT1 inhibition induced by DBC1 has been reported to accelerate apoptosis [21]. Although the function of DBC1 in the immune system has been reported in B cells and Tregs, its function in other immune cells remains largely blurry. DBC1 was reported to suppress B cell activation through NF‐kB signaling pathway [46]. DCs prime adaptive and innate immunity, and abnormal activation of DCs and the subsequent activation of T cells are key factors in the development of autoimmune diseases [47, 48]. In SLE, DC dysfunction plays a pivotal role in disease initiation and progression [49]. Currently, plentiful therapeutics for SLE treatment have achieved remarkable results in clinical trials by attenuating immune response through multiple targets, including Toll‐like receptor inhibitors, anti‐malarial agents (such as hydroxychloroquine that blocks TLR binding epitopes), blocking antibodies (such as IFN‐α, CD40, CD19, CD20, and FcRIIb), molecular agents (such as JAK1 inhibitor and STAT1 and STAT3 inhibitors), and autologous and allogeneic CD19 CAR‐T cells [50]. Though multiple therapeutic targets, SLE is still incurable and faces refractory and recurrent challenges. Our results showed STAT2, 3, 4, 5, and 6 were activated in DC upon LPS stimulation (Figure 1A), and DBC1 deficiency simultaneously decreased JAK1, 2, and 3 in Figure 5A. These results show that DBC1 regulates multiple JAK‐STAT signals in upstream, therefore targeting DBC1 may exert potent inhibition on JAK‐STAT signals rather than existing single JAK inhibitors. Thus, reshaping DC's function to ultimately tune immune response has a potential in SLE treatment, even cure.

In this study, we document that DBC1 deficiency partially decreases DCs apoptosis, but does not alter DC differentiation. Our findings reveal that DBC1 is transcriptionally and translationally upregulated in DCs following stimulation with LPS or ALD‐DNA, the most commonly used auto‐antigen to establish SLE mouse model [51]. DBC1 upregulation was associated with enhanced DC activation in response to stimulation, highlighting its involvement in the activation process of DCs. We generated a *Cd11c^cre^ Dbc1^−/−^* mouse strain to specifically knockout DBC1 on DCs, and these mice received subcutaneous immunization of ALD‐DNA, showing lower autoantibodies and milder LN pathological changes. In consideration that CD11c is also partially co‐expressed in macrophages, neutrophils, and B cells [52, 53, 54], we decided to strengthen the discovery that modulated DCs related to SLE pathogenesis by using another well‐established BMDC‐ALD‐DNA murine model. In the BMDC‐ALD‐DNA‐induced SLE model, DBC1‐deficient BMDCs were induced in vitro and stimulated by ALD‐DNA. Mice that received intravenous injection of these ALD‐DNA‐stimulated *Dbc1^−/−^* BMDCs also developed gentle LN accompanied with skewing Treg/Teff balance, showing enhanced Treg differentiation but decreased Tfh and Th2 polarization.

In SLE, the Treg function in inhibiting immune responses and Tfh has been well studied. Indeed, Tfh cells induce GCB to produce autoantibodies. The clinical trials targeting the enhancement of Treg and the depletion of Tfh have achieved clinical benefits [55, 56, 57]. In addition, Th2‐cell‐associated immunity also plays an important role in SLE, as Th2‐derived cytokines promote B cells activation, differentiation, and antibody production after the autoimmune antigen stimulation [36, 58]. In fact, Th2 cells secrete IL‐4 and IL‐5 that cause B‐cell isotype switching to IgE, but IgG production is unaffected [59, 60]. In SLE, dsDNA‐specific IgE was significantly higher in serum and deposited in kidneys, and the concentration of serum IgE correlated with disease severity [61, 62]. These self‐reactive IgE autoantibodies have been reported to amplify autoantibody production, which leads to LN [63]. A unique T helper cell subset, T follicular helper type 2 cells (Tfh2) that share signatures of both Tfh and Th2 cells (CXCR5^+^ PD1^+^ IL‐4^hi^ or GATA3^+^ BCL6^+^), was highly pathogenic in SLE [64]. In addition to increased IgG, the IgE level in SLE was markedly increased in GCB cells, serum, skin, and kidneys. Noticeably, the current study demonstrates that DBC1‐deficient DCs simultaneously restrained Th2 and Tfh in the murine model of SLE. That is the reason why we decided to investigate how DBC1 signal regulates DCs and affects T‐cell differentiation.

We noted that STATs (STAT 2, 3, 4, 5, 6) were significantly increased upon DC activation, while DBC1 deficiency impaired STAT5 expression both transcriptionally and translationally. The previous study reported that STAT5 in DCs is critical for Th2 development [38]. Normally, the exogenous cytokine TSLP binds TSLP receptor in DCs and activates intracellular STAT5. Since DC STAT5‐Th2 axis is well established and the specific role of Th2 in SLE development, we immediately investigated whether STAT5 is responsible for DC function change upon DBC1 deficiency. We separately overexpressed STAT5 in WT and *Dbc1^−/−^* DCs and stimulated these STAT5 overexpressed and normal WT/*Dbc1^−/−^* DCs with ALD‐DNA. In BMDC‐ALD‐DNA‐induced murine model of SLE, STAT5‐overexpressed *Dbc1^−/−^* DCs induced SLE symptoms as almost severe as WT DCs compared with *Dbc1^−/−^* DCs, suggesting that STAT5 is a predominant actor of DBC1 signal. Additionally, it was reported that STAT5 is not required for the development of DCs in vivo [38]; our findings further supported that DBC1 is firmly connected to STAT5 expression and also dispensable for DCs development. Collectively, this study provides the first evidence of the critical role of DBC1‐STAT5 pathway in the DC‐Th2 axis in SLE pathogenesis. This novel mechanistic insight highlights the potential role of DBC1 in the DCs and its influences in the development of SLE. Targeting DBC1 as the potential therapeutic strategy could be further explored in the future.

Although we demonstrated DBC1 modulates DC function through STAT5 signal, it is important to investigate how DBC1 mediates STAT5 expression. It has previously been reported that JAK3/STAT5 inhibitor BD750 induced tolerogenic dendritic cells by inhibiting DC maturation [65]; we therefore propose that DBC1‐deficient DCs obtained tolerogenic phenotypes and skewed effector T‐cell differentiation to Treg orientation. Indeed, in our murine model of LN, Treg cell population was upregulated, even though the mechanism of Treg induction needs to be further explored. It is also worth noting that Tfh induction was also reduced in *Dbc1^−/−^* DC SLE model. It is possible that *Dbc1^−/−^* DC directly suppresses Tfh or through enhancing Treg. Since DBC1 is regarded as a negative regulator of SIRT1 and it has been reported that SIRT1 activation promoted PD‐L1 gene expression [66], we deduce that DBC1 deficiency abolishes SIRT1 inhibition and therefore promotes B7‐H1. Upregulation of B7‐H1 in DCs was reported to support Treg differentiation through inhibiting CD28 signal and dampening effector T‐cell development [67]. Our RNA‐seq results showed DBC1 deficiency promotes TGF‐β production except for B7‐H1, which plays a critical role in Treg differentiation [68], and we therefore believe that DBC1‐deficient DCs regulate Treg through multiple signals. Our next plans will focus on clarifying other major target cells like Treg and Tfh cells upon DBC1 inhibition and exploring additional underlying mechanisms, thereby DBC1 regulates DCs. In human SLE, we found that DCs with lower *Dbc1* mRNA level showed low level of HLA‐DQA1, CD1C, and FCR1A, suggesting low expression of DBC1 presented a mild antigen‐presenting ability and leading to a tolerogenic phenotype.

Collectively, our results demonstrated that DBC1 in DCs influences the development of SLE through DBC1‐STAT5‐Th2 axis, highlighting its potential as a therapeutic target for modulating immune responses in SLE. This study explores the function of DBC1 in DCs and its influence on SLE for the first time. There are some limitations in this study. Since DBC1 has complicated roles in physiology and pathophysiology, developing a specific method to inhibit or degrade DBC1 may cause unexpected effects on other cells beyond DCs. Compared with other membrane surface molecules that only target a single signal pathway or overall B‐cell clearance that destroy humoral immunity, DBC1 inhibition may show a more powerful ability in regulating a series of immune responses of DCs, suppressing excessive activation of T‐cell immunity from initiation. Mechanistically, we have not yet fully detailed how DBC1 deficiency interferes STAT5 expression and the targeted proteins responsible for regulating STAT5. Although the great potential in targeting DBC1 in treating SLE, there is currently no available specific DBC1 inhibitor, which increases difficulties in clinical application. To address these concerns, further study is needed to clarify the DBC1 targeting molecules that define DC capacity and accordingly develop DBC1 inhibitors. Hence, it is a huge challenge and needs timing to develop the clinical translation targeting DBC1.

## 4 Materials and Methods

### 4.1 Mice

Female C57BL/6 and Balb/c mice aged 6–8 weeks were purchased from Beijing Vital River Laboratory Animal Technology Co., Ltd.; *Dbc1^−/−^* mice were bred by our group, *Cd11c^cre^* mice were generously provided by Gonghua Huang (Guangdong Medical University), DBC1^fl/fl^ mice were generously provided by Dr. Bing Li (Shanghai Jiao Tong University School of Medicine), *Cd11c^cre^ Dbc1^fl/fl^* mice were generated from cross breed of *Cd11c^cre^* mice and *Dbc1^fl/fl^* mice, and Balb/c‐*Il4^GFP^* mice were generously provided by Dr. Zhinan Yin (University of Jinan, Guangzhou). C57BL/6‐*Foxp3^GFP^* mice were kindly donated by Dr. Talil Chatilla from UCLA Medical School. Animals involved in this study were fed in the center of experimental animals of Shanghai Jiao Tong University (IACUC# 82371817‐01), Guangdong Medical Laboratory Animal Center (GDLAMI IACUC/T‐001), and Sun Yat‐sen University (IACUC# 2019022). The Institutional Animal Care and Use Committee at the above institutes has approved the use of animals and related animal experiments.

### 4.2 Bone Marrow–Derived Dendritic Cell Induction

As previously described [14], bone marrow cells were isolated from C57BL/6 or *Dbc1^−/−^* mice or *Cd11c^cre^ Dbc1^fl/fl^* mice, and then cultured with 50 ng/mL recombinant GM‐CSF (Peprotech) and 2.5 ng/mL rmIL‐4 (Peprotech) to induce the BMDC. To overexpress STAT5, a specific STAT5β lentiviral transfection was utilized to recover the STAT5 expression in BMDCs.

### 4.3 DCs Functional Experiments

Induced BMDCs were pre‐treated with different concentrations of LPS or ALD‐DNA, then the BMDCs were collected for the next western blot and quantitative PCR analyzation. As for the cytokines secretion and functional molecular expression of BMDCs, 2.5 µg/mL of LPS was added to the cultures for 24 h, BMDCs were collected for flow cytometry, and supernatants were collected for ELISA assay.

### 4.4 T Helper and Treg Polarization

To detect antigen‐presenting ability of WT and *Dbc1^−/−^* BMDCs, induced BMDCs were pre‐treated with 10 ng/mL LPS for 24 h to became semi‐mature DCs, then collected for T‐cell proliferation and polarization studies. CD4^+^ CD62L^+^ naïve T cells were isolated from the spleens from Balb/c mice, *Il‐4^GFP^* mice, or *Foxp3^GFP^* mice using a naïve T‐cell isolation kit (Stemcell). Splenocytes from Balb/c mice were incubated in a nylon wool column for 50 min and non‐adhere enriched T cells were collected for the proliferation assay. Enriched T cells were then labeled with 1 µM of 5(6)‐carboxyfluorescein diacetate succinimidyleste (CFSE) using the CellTraceTM CFSE cell proliferation kit (Thermo Fisher Scientific). The CFSE‐labeled T cells were cocultured with WT or *Dbc1^−/−^* BMDCs, and T‐cell proliferation was assessed after 72 h by flow cytometry. For Th1, Th2, Th17, and Treg cell polarization, semi‐mature WT or *Dbc1^−/−^* DCs were treated with mitomycin C and then cocultured with naïve CD4^+^ T cells from C57BL/6, *Il‐4^GFP^*, or *Foxp3^GFP^* mice in addition with T helper and Treg cell polarizing antibodies and cytokines for 72 h. Th1 polarization condition contained 1 µg/mL of anti‐CD3e, 1 µg/mL of anti‐CD28, 5 µg/mL anti‐IL4, and 10 ng/mL of rmIL‐12. Th2 polarization condition contained 1 µg/mL of anti‐CD3e, 1 µg/mL of anti‐CD28, 5 µg/mL, anti‐IFN‐γ, 5 µg/mL anti‐IL‐12, and 10 ng/mL of rmIL‐4. Th17 polarizing condition contained 1 µg/mL of anti‐CD3e, 1 µg/mL of anti‐CD28, 5 µg/mL, anti‐IFN‐γ, 1 µg/mL of anti‐CD3e, 1 µg/mL of anti‐CD28, 5 µg/mL of anti‐IFN‐γ, 5 µg/mL of anti‐IL‐12, 5 µg/mL of anti‐IL‐4, 5 ng/mL of rmTGF‐β, and 20 ng/mL of rmIL‐6. Treg polarizing condition contained 1 µg/mL of anti‐CD3e, 1 µg/mL of anti‐CD28, 5 ng/mL of rmTGF‐β, and 50 IU/mL of rmIL‐2. For cytokine detection, polarized Th1 and Th17 cells were stimulated with 50 ng/mL of phorbol 12‐myristate 13‐acetate (PMA) and 500 ng/mL of ionomycin for 5 h, and brefeldin A was added 1 h later than PMA and ionomycin to stimulate for rest 4 h. Flow cytometry was used to analyzed the IFN‐γ, IL‐17a, IL‐4^GFP^, or Foxp3^GFP^ ratio.

### 4.5 Lupus Model

For conventional mouse model of SLE [69], splenocytes from Balb/c mice were stimulated with LPS and concanavalin A for 6 days and collected to extract ALD‐DNA. Note that 100 µg/mL of ALD‐DNA was emulsified with complete Freund's adjuvant (CFA) in the volume of 1:1, and 100 µL of the emulsion was subcutaneously injected to the back of C57/BL6 mouse. At week 2 and week 4 post‐immunization, 100 µg/mL of ALD‐DNA was emulsified with incomplete Freund's adjuvant (IFA) in the volume of 1∶1, and mice received subcutaneous injection of the emulsion at a dose of 100 µL/mouse.

Mice sera were collected at Week 0, 4, 8, and 12 for Elisa assay of IgG, IgG1, IgG2a, and IgG2b. At the end of experiments, renal pathology and immune response were examined. HE (to assess lymphocyte infiltration), Masson (to assess renal fibrosis in which collagenous fiber showed blue), and PAS (periodic acid‐Schiff, to detect glycogen and other polysaccharide substances showing purple‐red) stainings were utilized to evaluate renal pathology. Deposition of IgG and complement C3 in kidneys was analyzed by immunofluorescence. DC phenotype was analyzed using flow cytometry.

For BMDC‐ALD‐DNA induced mouse model of SLE, as previously described [14], BMDCs were incubated with ALD‐DNA (activated lymphocytes derived DNA), which is produced as previously described [69] for 24 h. BMDC‐ALD‐DNA (1 × 10^6^ cells/mouse) in 200 µL of PBS was intravenously transferred into recipient C57BL/6 mice. Proteinuria was detected at different time points using semiquantitative Albustix paper (Gaoerbao). Sera of mice at Week 0, 2, 4, 8,12, 20, and 24 post immunization were collected, and IgG, IgG1, IgG2a, and IgG2b were measured using ELISA assay.

### 4.6 Flow Cytometry

Cells were resuspended in pre‐cool PBS containing 2% FBS and then stained with antibodies against CD11c, CD80, CD86, MHC II, CD69, CD40, CD163, CD25, B7‐H1, PD‐1, CD3, CD4, CD8, PD‐1, CD11b, Gr‐1, F4/80, FcR, CD138, CXCR5, B220, CD5, IgD, GL7s, IgM, and IgG (Biolegend). Co‐staining of intracellular cytokines IFN‐γ, IL‐17a, and intranuclear Foxp3 was conducted after stimulation with PMA and ionomycin for 4 h, following a standard protocol (BD Biosciences). The results were acquired using a BD FACS Fortessa flow cytometer and analyzed by FlowJo software.

### 4.7 Apoptosis Analyzation

BMDCs were collected after the induction culture condition, then the DCs were subjected to apoptosis analysis according to Annexin V‐APC/PI Apoptosis Kit (MULTI SCIENCE).

### 4.8 ELISA

Sera from lupus mice were collected at certain time points after the injection of BMDC‐ALD‐DNA to the recipient mice, and anti‐dsDNA antibodies were monitored using the semiquantitative method as described in the previous research [14]. The supernatants from LPS‐stimulated DCs were collected to detect the cytokines including TNF‐α, IFN‐γ, IFN‐β, CCL7, IL‐10, PGE2, IL‐6, IL‐4, and IL‐12 by an ELISA kit (Biolegend).

### 4.9 Quantitative Polymerase Chain Reaction

Total RNA extracted from BMDCs was performed using the RNeasy mini kit (Omega), and cDNA was generated by using a PrimeScriptTM RT‐PCR kit (TAKARA). The mRNA expression was quantified by using an SYBP Premix Ex TaqTM II Kit (TAKARA). The samples were analyzed in triplicate, and the relative expression of the target genes was determined by normalizing each target gene's to β‐actin through the 2^−ΔΔCt^ method.

### 4.10 Western Blot

BMDCs were collected and lysed with lysing buffer (Sigma) for 30 min on ice. Protein extracts were centrifuged at 4°C for 10 min, and the supernatants were harvested for concentration determination using a BCA Protein Concentration Assay kit (Beyotime).

Concentration‐adjusted protein extracts were subjected to vertical electrophoresis with 10% polyacrylamide‐SDS gels and horizontal electrophoresis onto nitrocellulose membranes. The membranes were blocked with Tris buffered saline containing 5% bovine serum albumin and incubated with antibodies against Bcl6 and β‐actin or GAPDH (Cell Signaling Technology), and then incubated with an HRP‐conjugated secondary antibody (Cell Signaling Technology). The blots were normalized to β‐actin or GAPDH.

### 4.11 Immunohistochemical Analysis of Kidneys

For immunohistochemical kidney analysis, experiments were terminated at week 24 after subcutaneous ALD‐DNA immunization or at Week 20 after the injection of BMDC‐ALD‐DNA. Kidneys were collected at the end of experiments and fixed in formalin for later paraffin section. Then these kidney sections were executed for the staining of hematoxylin and eosin (HE), Masson's trichrome, and PAS. Sections were observed with a fluorescence microscope (ZEISS, Germany). The severity of nephritis was evaluated by the pathological lesions (PL) using a standard HE scoring system (0–4: 0 for normal, 1 for mild glomerular mesangium hyperplasia, 2 for moderate mesangium hyperplasia, 3 for the formation of glomerular lobular plus the incrassation of basement membrane, and 4 for the formation, sclerosis, tubular atrophy, and casts of glomerular crescents). The level of interstitial fibrosis (IF) was assessed by Masson scoring (0–4: 0 for normal, 1 for damage area less than 25%, 2 for damage area between 25% and 50%, 3 for damage area between 50% and 75%, and 4 for damage area more than 75%).

The level of GD was assessed by PAS scoring (0–4: 0 for normal, 1 for GD area less than 25%, 2 for GD area between 25% and 50%, 3 for GD area between 50% and 75%, 4 for GD area more than 75%, and 5 for complete sclerotic glomeruli), in which three random fields at 400× magnification were chosen from five mice in each group. The scale bars were 50 µm at 400× magnification.

### 4.12 Immunofluorescence Analysis of Kidneys

Kidneys of lupus mice were collected at the end of experiments and subjected to frozen sections. These frozen sections of kidneys were fixed in 100% acetone and 1% paraformaldehyde for later staining of rabbit anti‐mouse C3 (Abcam) and goat anti‐mouse IgG (Abcam). The ZEISS microscope from Germany was used to analyze the fluorescence of these sections. The mean fluorescence intensity (MFI) of IgG and C3 represents the deposition level in kidneys (Image J). The scale bars were 50 µm at 400× magnification.

### 4.13 Statistics Analysis

Statistical analyses involved in this study were analyzed by GraphPad Prism. Comparison between the two groups was performed by unpaired two‐tailed *t* tests. One‐way or two‐way analysis of variance (ANOVA) was used to analyze the differences among multiple groups. Statistical significance was represented by *p* value < 0.05. Values represent the means ± SEM of at least three independent experiments: **p < 0.005, ***p < 0.0005, and ****p < 0.0001.

## Author Contributions

S.G.Z., D.W., and Z.X.X. designed the research. Z.X.X., Y.L., and R.L. performed the experiments. Q.F., X.H., and J.W. provided assistances and guidance on experiments. C.H. contributed to software. Z.X.X. and S.G.Z. wrote the original manuscript. S.G.Z. and N.O. contributed to editing the manuscript. The final version of this manuscript was approved by all authors.

## Ethics Statement

Animal studies in this work were approved by institutional committees of the Shanghai Jiao Tong University (IACUC# 82371817‐01), the Guangdong Medical Laboratory Animal Center (GDLAMI IACUC/T‐001), and the Sun Yat‐sen University (IACUC# 2019022).

## Conflicts of Interest

Song Guo Zheng is an editorial board member of *MedComm*. Song Guo Zheng was not involved in the journal's review or decisions related to this manuscript. The authors declare no conflict of interest.

## Supporting information




**Figure S1**: DBC1 participated the activation but not development of dendritic cell. A. The genetic identification of *Cd11c^cre^ Dbc1^fl/fl^* mice. B. The ratio of B cell, T cell, CD4^+^ T cell, CD8^+^ T cell, macrophage, NK cell and neutrophil of different tissues from the *Cd11c^cre^* and *Cd11c^cre/cre^ Dbc1^fl/fl^* mice. C. The DC characters of different tissues from the *Cd11c^cre^* and *Cd11c^cre/cre^ Dbc1^fl/fl^* mice. The results are presented as the mean ± s.e.m. from three separate experiments. ns indicates no significance, **p < 0.005, ***p < 0.0005, and ****p< 0.0001 using nonparametric Mann–Whitney tests.
**Figure S2**: Immune changes of the SLE model mice. A. The CD40, MHCII and CCR7 expression of DC from the SLE model mice. B. The B cell and T cell frequency of the SLE model mice. C. The functional markers of B cells of the SLE model mice. The results are presented as the mean ± s.e.m. from three separate experiments. ns indicates no significance, **p < 0.005, ***p < 0.0005, and ****p < 0.0001 using nonparametric Mann–Whitney tests.
**Figure S3**: Immune changes of the SLE model mice. A. The development of dendritic cells in vivo from the WT or *Dbc1^−/−^* mouse. B. The production of TNF‐α, IFN‐γ, IFN‐β, CCL17 and IL‐10 from the WT or *Dbc1^−/−^* BMDC. C. The maturation and activation markers of WT or *Dbc1^−/−^* DCs after the stimulation of LPS. D. The Th1 and Th17 cell polarization under the sitmulation of WT or *Dbc1^−/−^* DCs. The results are presented as the mean ± s.e.m. from three separate experiments. ns indicates no significance, **p < 0.005, ***p < 0.0005, and ****p < 0.0001 using nonparametric Mann–Whitney tests.
**Figure S4**: Stat5b over expression plasmid.
**Figure S5**: STAT5β over expression rescued the *Dbc1^−/−^* BMDCs‐ALD‐DNA induced amelioration of SLE syndrome. WT and *Dbc1^−/−^* BMDCs were transfected with lentivirus to over express STAT5 to obtain WT *Stat5^ov^* and *Dbc1^−/−^ Stat5^ov^* BMDCs. WT, *Dbc1^−/−^*, WT *Stat5^ov^* and *Dbc1^−/−^ Stat5^ov^* BMDCs were separately stimulated by ALD‐DNA for 24 h, and were than intravenously injected to C57/BL6 mice to establish murine model of SLE. A. The anti‐ds‐DNA IgG1 and IgG2a of WT, *Dbc1^−/−^*, WT *Stat5^ov^* and *Dbc1^−/−^ Stat5^ov^* BMDC‐injected mice; B‐D. The immune cell analysis of spleen (B‐C) and lymph nodes (D) from mice of 4 groups. The results are presented as the mean ± s.e.m. from three separate experiments. ns indicates no significance, **p < 0.005, ***p < 0.0005, and ****p < 0.0001 using nonparametric Mann–Whitney tests.
**Figure S6**: DBC1 deficiency lowered proinflammatory characters of DCs. Bone marrow cells from wild type and *Dbc1^−/−^* mice were used to induced BMDCs under the condition of 50 ng/mL GM‐CSF and 2.5 ng/mL IL‐4 for 6 days. Adhere DC clusters were collected and separated into unstimulated groups and LPS‐stimulated groups. Wild type and *Dbc1^−/−^* BMDCs were stimulated with 100 ng/mL for 24 h and then collected for RNA‐seq. A. PCA analysis to show cluster of all samples. B. Volcano plot of Differential expressed genes comparing LPS‐stimulated *Dbc1^−/−^* BMDCs with LPS‐stimulated wild type BMDCs. C. KEGG analysis comparing LPS‐stimulated *Dbc1^−/−^* BMDCs with LPS‐stimulated wild type BMDCs. D. Reactome analysis comparing LPS‐stimulated *Dbc1^−/−^* BMDCs with LPS‐stimulated wild type BMDCs. E. Gene Set Enrichment Analysis (GSEA) analysis to show overall changes of specific gene sets. Absolute value of Normalized Enrichment Score (NES)>1 represents significant enrichment. False discovery rate (FDR)<0.25 represents statistical significance.
**Figure S7**: DBC1 expression in healthy controls and SLE patients. A. Quantitative analysis of *Tslp*, *Tslpr* and *Il7r* mRNA expression of WT and *Dbc1^−/−^* BMDCs by RNA‐seq. B. Bulk RNA‐seq results of DBC1 mRNA level in DCs from healthy controls and SLE patients (E‐GEAD‐397). C. Single‐cell RNA‐seq results of DBC1 mRNA level in DCs from healthy controls and SLE patients (GSE135779). D. Single‐cell RNA‐seq results of DBC1 mRNA level in DCs from healthy controls and SLE patients (GSE174188). ns means no significance.

## Data Availability

Data will be available upon request.
