# Supplementary material for: Regulation and Mechanism of Deleted in Breast Cancer‐1 on Dendritic Cell Function in Systemic Lupus Erythematosus
Source: MedComm (2020). 2026 Jan 14;7(1):e70581. doi: 10.1002/mco2.70581 (PMC12801396; doi:10.1002/mco2.70581)
Supplement: Supplementary file 1 — Figure S1: DBC1 participated the activation but not development of dendritic cell. A. The genetic identification of Cd11ccre Dbc1fl/fl mice. B. The ratio of B cell, T cell, CD4+ T cell, CD8+ T cell, macrophage, NK cell and neutrophil of different tissues from the Cd11ccre and Cd11ccre/cre Dbc1fl/fl mice. C. The DC characters of different tissues from the Cd11ccre and Cd11ccre/cre Dbc1fl/fl mice. The results are presented as the mean ± s.e.m. from three separate experiments. ns indicates no significance, **p < 0.005, ***p < 0.0005, and ****p< 0.0001 using nonparametric Mann–Whitney tests. Figure S2: Immune changes of the SLE model mice. A. The CD40, MHCII and CCR7 expression of DC from the SLE model mice. B. The B cell and T cell frequency of the SLE model mice. C. The functional markers of B cells of the SLE model mice. The results are presented as the mean ± s.e.m. from three separate experiments. ns indicates no significance, **p < 0.005, ***p < 0.0005, and ****p < 0.0001 using nonparametric Mann–Whitney tests. Figure S3: Immune changes of the SLE model mice. A. The development of dendritic cells in vivo from the WT or Dbc1−/− mouse. B. The production of TNF‐α, IFN‐γ, IFN‐β, CCL17 and IL‐10 from the WT or Dbc1−/− BMDC. C. The maturation and activation markers of WT or Dbc1−/− DCs after the stimulation of LPS. D. The Th1 and Th17 cell polarization under the sitmulation of WT or Dbc1−/− DCs. The results are presented as the mean ± s.e.m. from three separate experiments. ns indicates no significance, **p < 0.005, ***p < 0.0005, and ****p < 0.0001 using nonparametric Mann–Whitney tests. Figure S4: Stat5b over expression plasmid. Figure S5: STAT5β over expression rescued the Dbc1−/− BMDCs‐ALD‐DNA induced amelioration of SLE syndrome. WT and Dbc1−/− BMDCs were transfected with lentivirus to over express STAT5 to obtain WT Stat5ov and Dbc1−/− Stat5ov BMDCs. WT, Dbc1−/− , WT Stat5ov and Dbc1−/− Stat5ov BMDCs were separately stimulated by ALD‐DNA for 24 h, and were than in [file MCO2-7-e70581-s001.docx]

**Regulation and mechanism of Deleted in Breast Cancer-1 on dendritic cell function in systemic lupus erythematosus**

**Running Title: DBC1 regulates dendritic cells in SLE**

Zexiu Xiao^1,2,3#^, Rongzhen Liang^2#^, Yan Liu^3,4#^, Changyuan Huang^5^, Qiannan Fang^2,7^, Xiaojiang Hu^3^, Julie Wang^2^, Nancy Olsen^6^, Dehua Wu^1*^, Song Guo Zheng^2*^

^1^Department of Anesthesiology, Songjiang Hospital affiliated to the Shanghai Jiao Tong University School of Medicine, Shanghai 201600, China

^2^Department of Immunology, the School of Cell and Gene Therapy, Songjiang Research Institute and Songjiang Hospital affiliated to the Shanghai Jiao Tong University School of Medicine, Shanghai 201600, China

^3^Department of Clinical Immunology, the Third Affiliated Hospital of Sun Yat-sen University, Guangzhou, 510630, China

^4^Department of Rheumatology, the Third Affiliated Hospital of Sun Yat-sen University, Guangzhou, 510630, China

^5^The First Dongguan Affiliated Hospital, Guangdong Medical University, Dongguan 523710, China

^6^Department of Medicine, Penn State College of Medicine, Hershey, PA, 17033, United States

^7^Medical Research institute, Guangdong Provincial People's Hospital (Guangdong Academy of Medical Science), Southern Medical University, Guangzhou, 510080, China

^#^These authors contributed equally

^*^Correspondence to Song Guo Zheng, MD, PhD, Email: [Song.Zheng@shsmu.edu.cn](mailto:Song.Zheng@shsmu.edu.cn) and Dehua Wu, MD, Email: [wudehua74@163.com](mailto:wudehua74@163.com)

**Supplementary figures
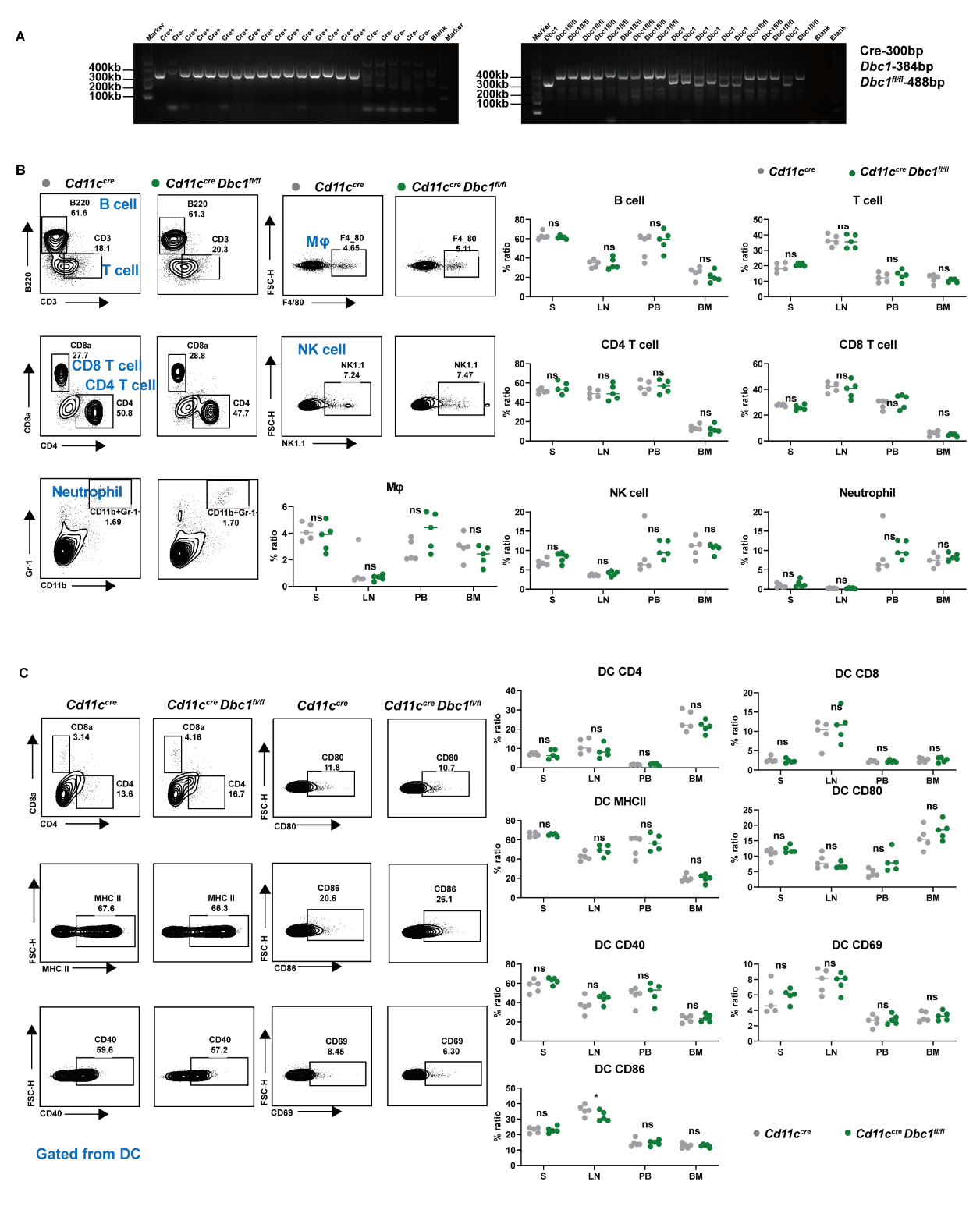
**

**Figure S1. DBC1 participated the activation but not development of dendritic cell.**

**A**. The genetic identification of *Cd11c^cre^ Dbc1^fl/fl^* mice. **B**. The ratio of B cell, T cell, CD4^+^ T cell, CD8^+^ Tcell, macrophage, NK cell and neutrophil of different tissues from the *Cd11c^cre^* and *Cd11c^cre/cre^ Dbc1^fl/fl^* mice. **C**. The DC characters of different tissues from the *Cd11c^cre^* and *Cd11c^cre/cre^ Dbc1^fl/fl^* mice. The results are presented as the mean ± s.e.m. from three separate experiments. ns indicates no significance, ***p* < 0.005, ****p* < 0.0005, and *****p*< 0.0001 using nonparametric Mann–Whitney tests.


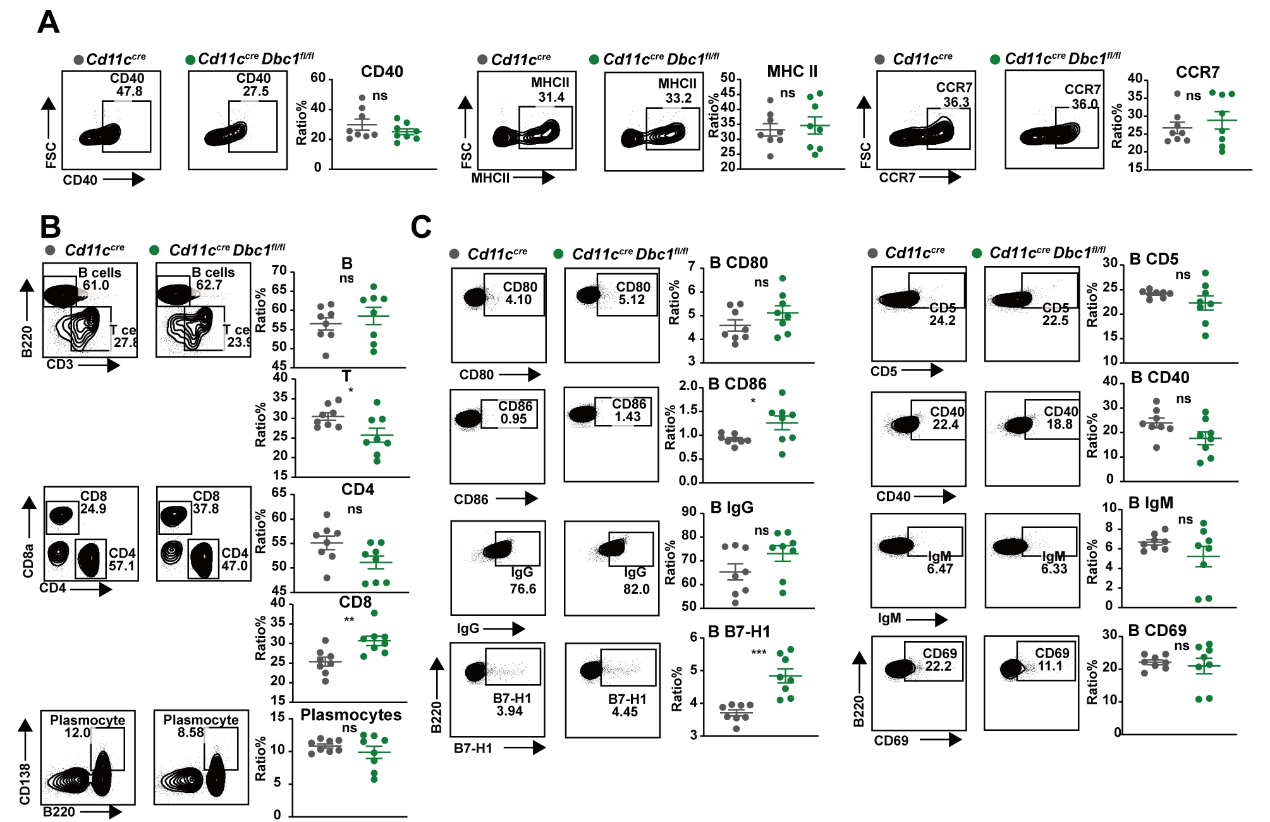


**Figure S2. Immune changes of the SLE model mice.**

**A.** The CD40, MHCII and CCR7 expression of DC from the SLE model mice. **B**. The B cell and T cell frequency of the SLE model mice. **C**. The functional markers of B cells of the SLE model mice. The results are presented as the mean ± s.e.m. from three separate experiments. ns indicates no significance, ***p* < 0.005, ****p* < 0.0005, and *****p* < 0.0001 using nonparametric Mann–Whitney tests.


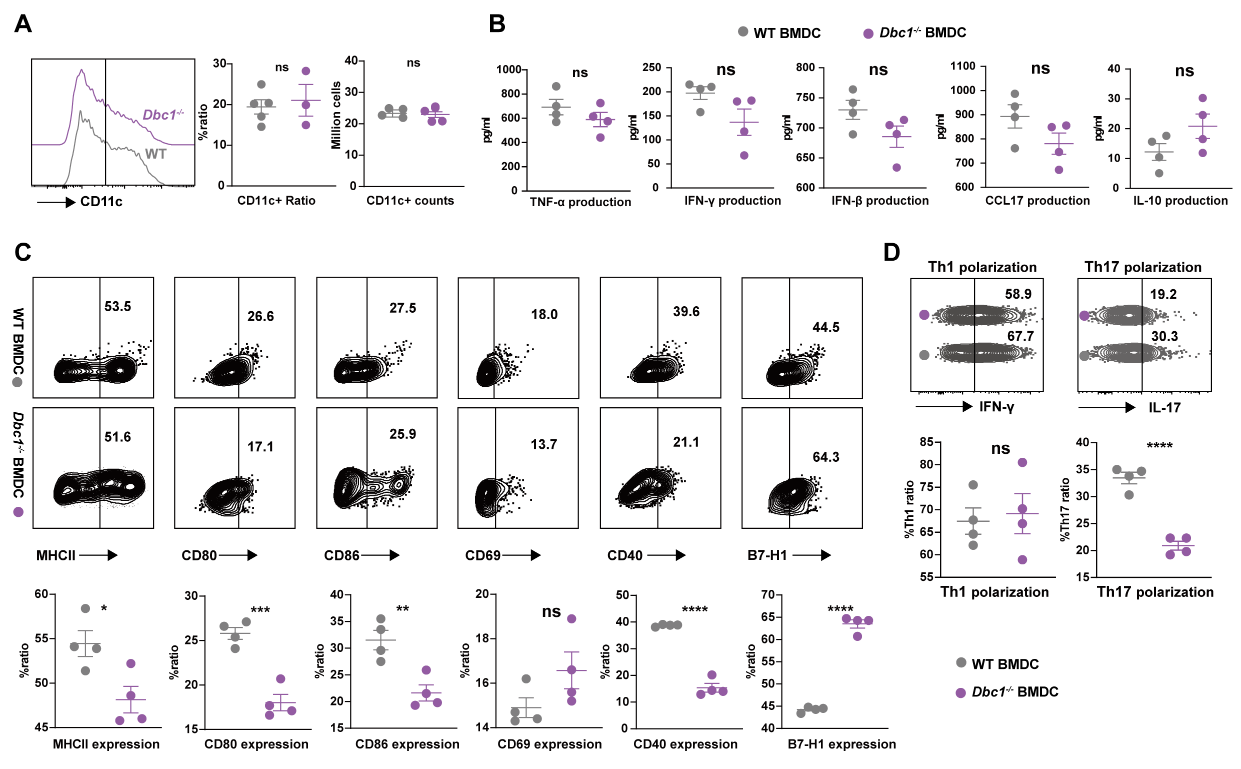


**Figure S3. Immune changes of the SLE model mice.**

**A.** The development of dendritic cells in vivo from the WT or *Dbc1^-/-^* mouse. **B**. The production of TNF-α, IFN-γ, IFN-β, CCL17 and IL-10 from the WT or *Dbc1^-/-^* BMDC. **C.** The maturation and activation markers of WT or *Dbc1^-/-^* DCs after the stimulation of LPS. D. The Th1 and Th17 cell polarization under the sitmulation of WT or *Dbc1^-/-^* DCs. The results are presented as the mean ± s.e.m. from three separate experiments. ns indicates no significance, ***p* < 0.005, ****p* < 0.0005, and *****p* < 0.0001 using nonparametric Mann–Whitney tests.


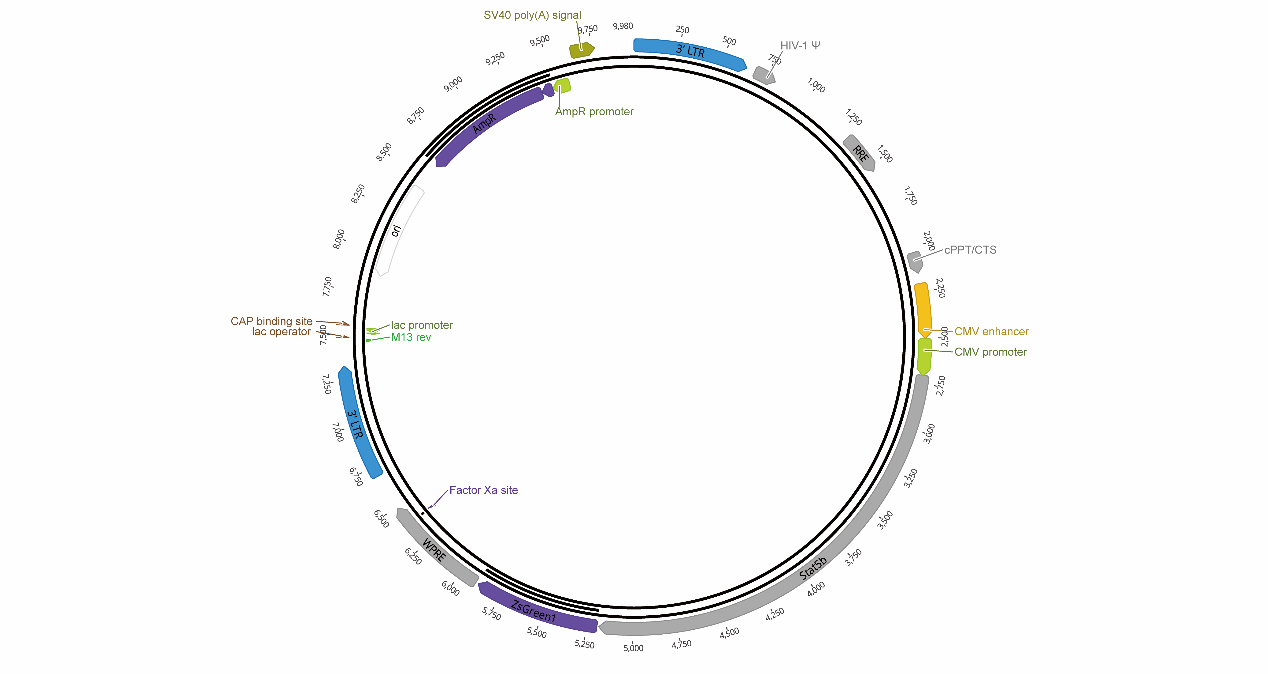


**Figure S4. Stat5b over expression plasmid**


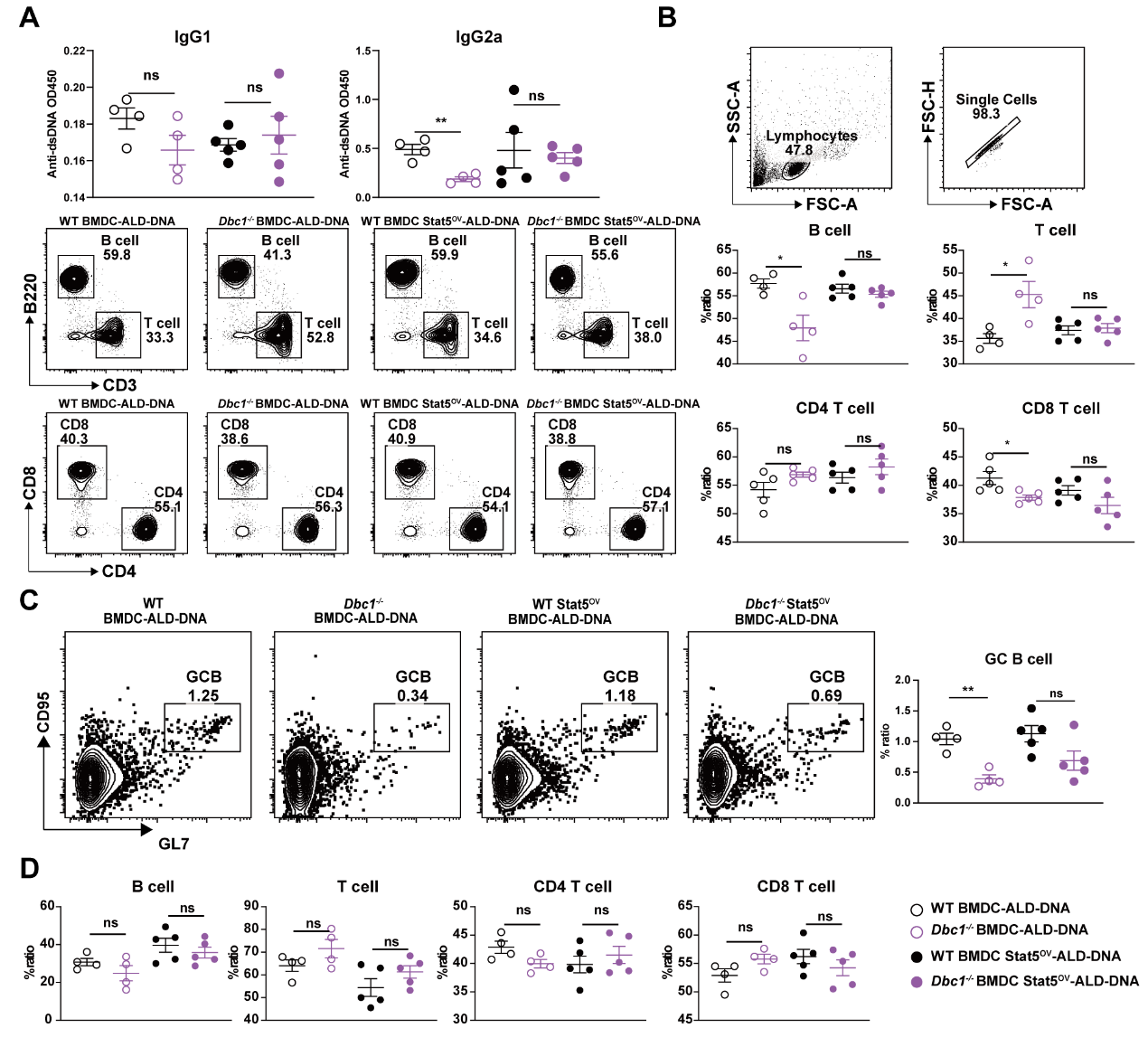


**Figure S5. STAT5β over expression rescued the *Dbc1^-/-^* BMDCs-ALD-DNA induced amelioration of SLE syndrome.**

WT and *Dbc1^-/-^* BMDCs were transfected with lentivirus to over express STAT5 to obtain WT *Stat5^ov^* and *Dbc1^-/-^ Stat5^ov^* BMDCs. WT, *Dbc1^-/-^*, WT *Stat5^ov^* and *Dbc1^-/-^ Stat5^ov^* BMDCs were separately stimulated by ALD-DNA for 24 hours, and were than intravenously injected to C57/BL6 mice to establish murine model of SLE. **A.** The anti-ds-DNA IgG1 and IgG2a of WT, *Dbc1^-/-^*, WT *Stat5^ov^* and *Dbc1^-/-^ Stat5^ov^* BMDC-injected mice; **B-D.** The immune cell analysis of spleen **(B-C)** and lymph nodes **(D)** from mice of 4 groups. The results are presented as the mean ± s.e.m. from three separate experiments. ns indicates no significance, ***p* < 0.005, ****p* < 0.0005, and *****p* < 0.0001 using nonparametric Mann–Whitney tests.


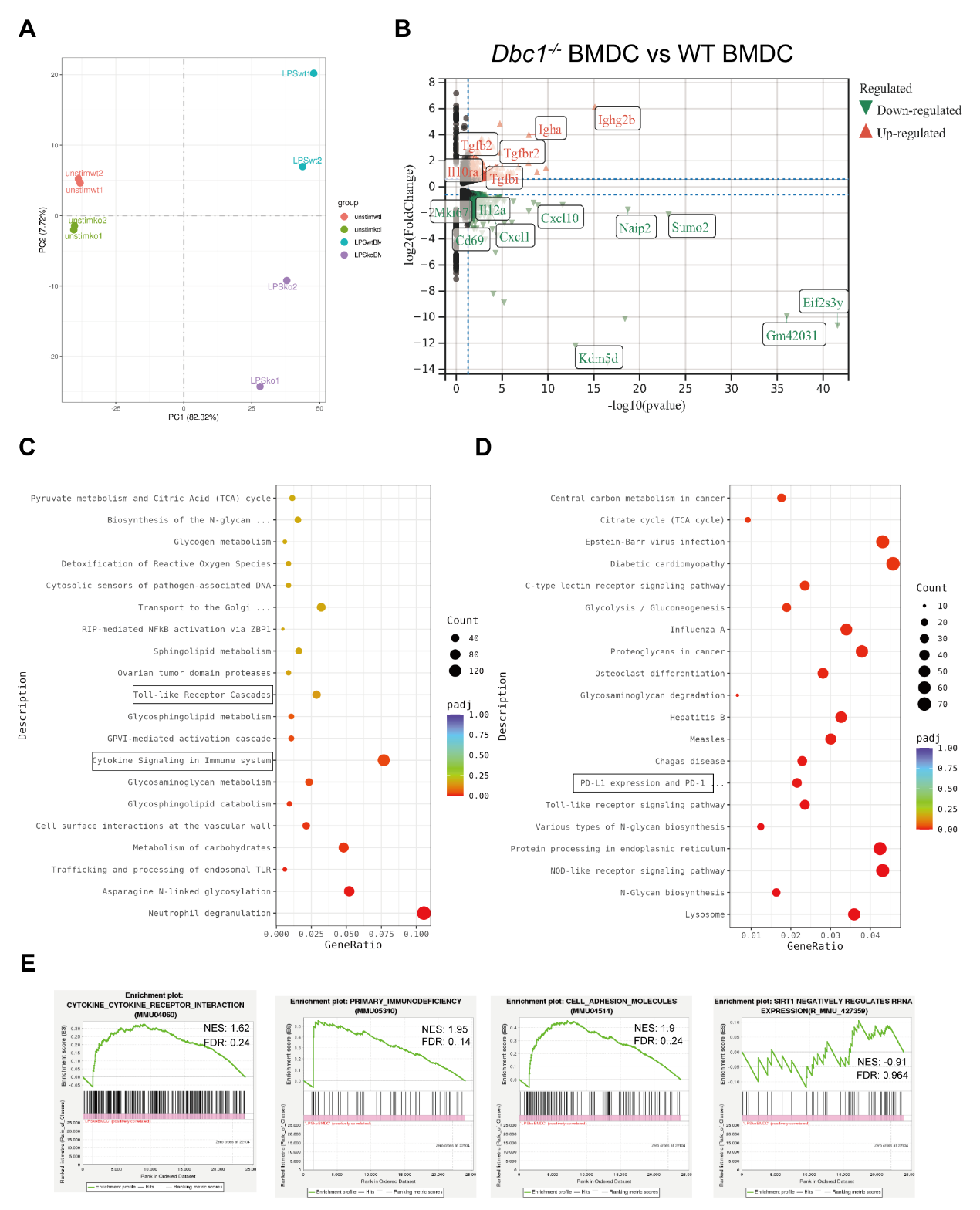


**Figure S6. DBC1 deficiency lowered proinflammatory characters of DCs.** Bone marrow cells from wild type and *Dbc1^-/-^* mice were used to induced BMDCs under the condition of 50 ng/mL GM-CSF and 2.5 ng/mL IL-4 for 6 days. Adhere DC clusters were collected and separated into unstimulated groups and LPS-stimulated groups. Wild type and *Dbc1^-/-^* BMDCs were stimulated with 100 ng/mL for 24 hours and then collected for RNA-seq.

**A**. PCA analysis to show cluster of all samples. **B**. Volcano plot of Differential expressed genes comparing LPS-stimulated *Dbc1^-/-^* BMDCs with LPS-stimulated wild type BMDCs. **C**. KEGG analysis comparing LPS-stimulated *Dbc1^-/-^* BMDCs with LPS-stimulated wild type BMDCs. **D**. Reactome analysis comparing LPS-stimulated *Dbc1^-/-^* BMDCs with LPS-stimulated wild type BMDCs. **E**. Gene Set Enrichment Analysis (GSEA) analysis to show overall changes of specific gene sets. Absolute value of Normalized Enrichment Score (NES)>1 represents significant enrichment. False discovery rate (FDR)<0.25 represents statistical significance.


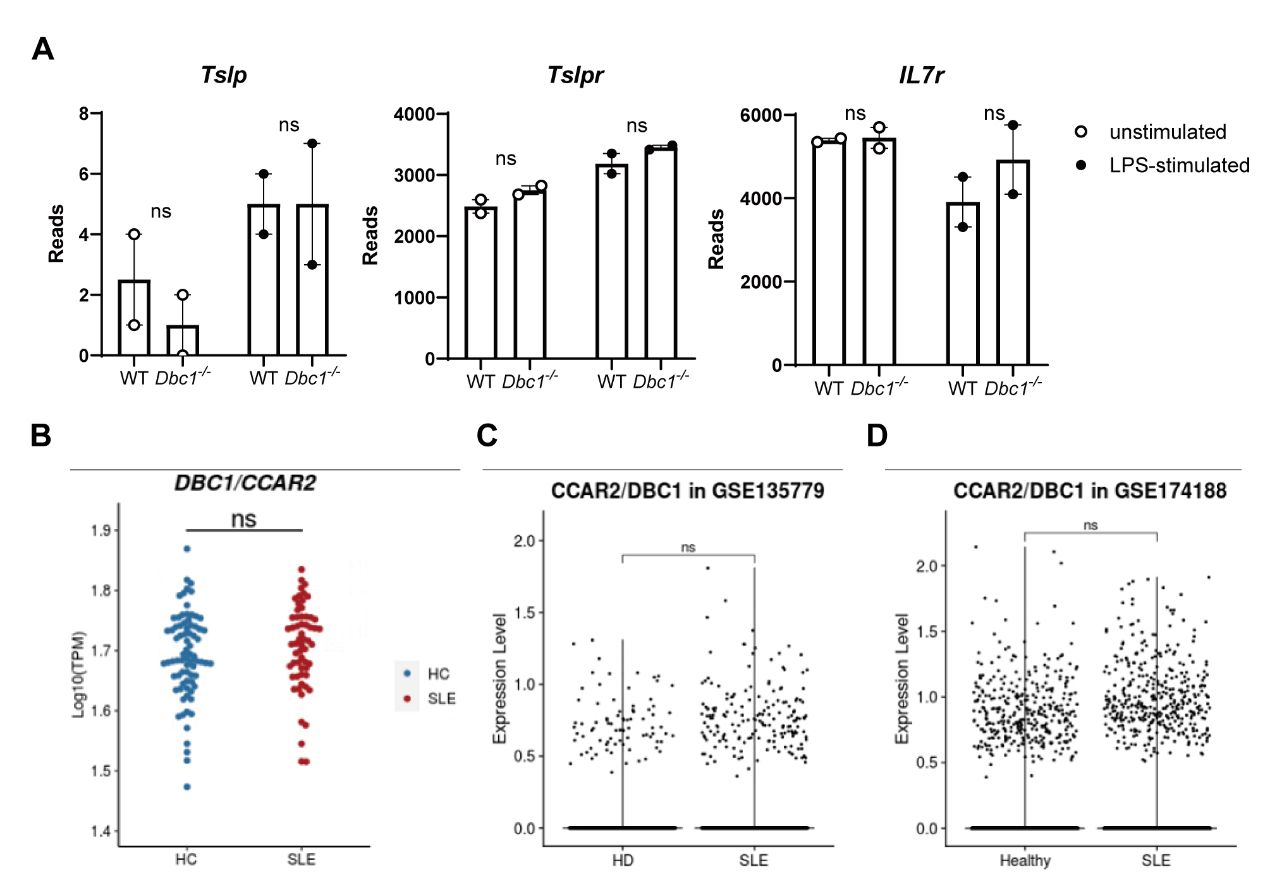


**Figure S7. DBC1 expression in healthy controls and SLE patients.**

**A.** Quantitative analysis of *Tslp*, *Tslpr* and *Il7r* mRNA expression of WT and *Dbc1^-/-^* BMDCs by RNA-seq. **B**. Bulk RNA-seq results of DBC1 mRNA level in DCs from healthy controls and SLE patients (E-GEAD-397). **C**. Single-cell RNA-seq results of DBC1 mRNA level in DCs from healthy controls and SLE patients (GSE135779). **D**. Single-cell RNA-seq results of DBC1 mRNA level in DCs from healthy controls and SLE patients (GSE174188). ns means no significance.
